# Fascia as a Functional System in Health and Disease: From Fundamental Biology to Assessment and Targeted Interventions

**DOI:** 10.3390/ijms27135871

**Published:** 2026-06-29

**Authors:** Hao Huang, Lei Chen, Yitian Lai, Wu Li, Jiangshan Li

**Affiliations:** College of Acupuncture, Moxibustion, Tuina and Rehabilitation, Hunan University of Chinese Medicine, Changsha 410208, China; 20252137@stu.hnucm.edu.cn (H.H.); 20223789@stu.hnucm.edu.cn (L.C.); laiyt@stu.hnucm.edu.cn (Y.L.); 003976@hnucm.edu.cn (W.L.)

**Keywords:** fascia, fascial function, assessment, targeted intervention, chronic pain, fascial disorders, musculoskeletal system

## Abstract

Fascia is increasingly recognized as a dynamic functional system. It can actively sense, transmit, and regulate mechanical, sensory, and metabolic signals. Why does fascia play such a critical role in chronic pain and movement disorders? Researchers are now rethinking the pathophysiological mechanisms underlying this role. Previous systematic reviews have typically focused primarily on specific mechanisms or interventions. In contrast, this study takes a holistic view of fascial function. It integrates multiple physiological functions of the fascia: mechanical integration, sensory modulation, cellular and matrix remodeling, as well as metabolic and immune regulation. From the perspective of functional imbalance, we further explore the pathological mechanisms associated with the fascia. Building on this, we then focus on how to assess fascial function from multiple dimensions and on specific targeted interventions. For assessment, we have systematically compiled a set of multi-stage quantitative techniques. These include clinical palpation, ultrasound, and elastography, tissue mechanics testing, microdialysis, omics approaches, electrophysiological testing, and digital modeling. For interventions, we have listed a range of modulating approaches, such as manual therapy, exercise rehabilitation, dry needling and acupuncture, fascial injections, targeted drugs, and biotechnological materials derived from tissue engineering. This review summarizes a clinical decision-making framework guided by the assessment of fascial functional status. It emphasizes a systematic approach and links quantitative diagnosis with precise interventions. Additionally, it provides a literature synthesis for understanding fascial mechanisms and related disorders and offers a reference foundation for the field’s transition from empirical treatment to measurable, reproducible, and individualized practice.

## 1. Introduction

Fascia is a connective tissue that runs throughout the whole body. Traditionally, people viewed it mainly as a passive structure, something that provides mechanical support. That is why fascia has often been called the “white sheath of the muscles” [1]. But as research has advanced in recent years, our understanding of fascia has kept evolving. According to current knowledge, fascia forms a continuous, layered, and actively adaptive three-dimensional network. It also acts as a functional hub. From this hub, it can actively sense and transmit mechanical signals, help modulate the chemical microenvironment, and interact closely with neurovascular structures. Because of these abilities, fascia exerts a decisive influence on motor control, maintenance of homeostasis, and injury repair. In short, its essential functions go far beyond the traditional passive role [2].

Increasing evidence suggests that fascial structural and functional abnormalities are closely associated with many musculoskeletal pain conditions and movement disorders. Current evidence supports a bidirectional relationship in which initial tissue injury, abnormal mechanical loading, inflammation, or metabolic disturbances may induce fascial remodeling, while persistent fascial dysfunction can further amplify nociceptive signaling, impair force transmission, and contribute to chronic symptom maintenance [3,4]. Through this dynamic interaction, fascia may act as both a responder to pathological stress and an active participant in disease progression. Multiple mechanisms have been proposed to explain how fascial dysfunction participates in this process, including changes in the rheological properties of hyaluronic acid (HA), pathological activation of fibroblasts, and sensitization of nociceptors. Together, they create a microenvironment that promotes chronic pain maintenance and functional impairment [4,5,6]. Therefore, some clinical problems once thought to originate directly from muscles may actually arise from dysfunctions in the fascial system. Given this understanding, the clinical importance of fascia has become more and more prominent. Methods for assessing fascial function have also changed. They have moved from traditional palpation and symptom observation toward a multidimensional diagnostic system, one that now includes imaging diagnostics, biomechanics, and even the molecular and cellular levels [4]. Similarly, intervention strategies aimed at the fascial system are no longer limited to manual therapy. They have expanded to include rehabilitative exercises, injection therapies, and pharmacological modulation [7], showing the fascial system has broad potential as a therapeutic target.

Accordingly, this article takes fascial function as its central theme and systematically integrates the latest research findings within a logical framework encompassing physiology, pathology, diagnosis, and treatment. It particularly focuses on the latest progress in integrating the structural and functional basis of fascia, the mechanisms of functional disorders, quantitative assessment methods, and targeted intervention strategies. The aim is to establish a clinical decision-oriented framework for fascial function assessment and intervention, thereby providing a foundation for accurate diagnosis and optimized treatment of related diseases.

## 2. Overview of Physiological Functions of Fascia

### 2.1. Mechanics and Structural Functions

Fascia not only participates in the passive transmission of force but also actively perceives, adapts to, and guides biomechanical signals, serving as a crucial foundation for maintaining the body’s biomechanical integrity. Structurally, fascia forms a continuous force transmission system extending from the body surface to deep skeletal structures [8], and can be classified into superficial fascia, deep fascia, myofascia, and visceral fascia [3,9]. Superficial fascia is rich in elastic fibers, possesses excellent extensibility, and functions primarily through elastic adaptation and gliding [10]. Deep fascia is primarily composed of collagen fibers, characterized by a loose outer layer enclosing a dense central layer. This central layer can reversibly switch between wave-like and linear arrangements, exhibiting nonlinear mechanical properties similar to a spring, and primarily serves the function of transmitting tension [11]. Within the muscle, the myofascia is further subdivided into the epimysium, perimysium, and endomysium. While integrating and transmitting force from the entire muscle to individual muscle fibers in stages [12], these layers also form relatively independent mechanical units through the boundary properties of the fascia [13]. The epimysium is continuous with the tendon and ultimately integrates with the periosteum at the bone surface to form the osteotendinous attachment. This hierarchical fascia-muscle-tendon-bone organization provides a continuous structural pathway for force transmission and mechanical coupling across musculoskeletal tissues [14].

Based on this structure, fascia enables multidirectional force transmission: it facilitates continuous longitudinal conduction from superficial to deep layers, while dispersing tension laterally across muscle groups and joints through fascial chains [15]. Experimental studies indicate that 30% of muscle force is transmitted to distal regions via fascial structures [16], which explains referred pain and effects on distal structures. Consequently, fascia not only transmits force but also directionally guides and amplifies mechanical signals according to local functional demands, thereby shaping force to a certain extent [17]. Furthermore, findings such as the cervico-ocular fascial continuum suggest that fascial networks may mediate mechanical coupling across anatomical regions, thereby participating in the integration of sensory and motor functions [18]. These mechanical signals are ultimately converted into biological signals at the cellular level through mechanotransduction, thereby promoting adaptive tissue remodeling [8].

Fascia continuously senses and responds to the mechanical environment, and its mechanical state is primarily regulated by synergistic interactions between cells and the matrix. First, myofibroblasts generate tension via alpha-smooth muscle actin(α-SMA), thereby establishing coordinated intercellular responses [19]. Second, the synthesis and hydration status of HA can rapidly alter the lubricity between fascial layers and the overall stiffness of the fascia [3,19]. Based on this, mechanical stimuli are transmitted intracellularly via the “extracellular matrix(ECM)-integrin-cytoskeleton-nucleus” pathway, enabling cells to perceive various mechanical inputs and translate them into specific responses [20]. The newly proposed “calcium-hyaluronan axis” theory further elucidates how fascia can modulate matrix components through mechanosensitive ion channels [21]. Therefore, fascia responds differently to various types of stimulation, such as tension or shear forces, thereby participating in the regulation of the local mechanical environment.

Furthermore, disagreement still remains over how to define fascia and where its boundaries lie. Strictly defined, fascia is limited to dense, irregular connective tissue, and adipose and loose connective tissues are considered as accessory structures [22]. However, the concept of fascia has been expanded into a continuum through a more comprehensive perspective. That continuum includes solids (ligaments, fascia, etc.), fluids (blood and lymph), and even so-called holographic fascia (electromagnetic information networks). According to this view, fascial function is determined by fluid flow, but not by mechanical deformation. It also emphasizes the role of shear forces generated by interstitial fluid flow in mechanical signal transmission [23,24]. These differing viewpoints are reflected not only in anatomical definitions. They also reflect the fact that our understanding of fascia’s mechanical functions and mechanisms is still evolving, and related issues still require further research.

### 2.2. Sensory and Signaling Functions

Fascia also functions as a highly innervated system that is involved in sensory perception and signal integration. It takes part in complex processes like proprioception, pain perception, and even emotional regulation. These sensory functions are made possible mainly by a rich and specialized network of nerves. Anatomical studies have shown that the number of sensory nerve endings within fascia is much higher than that found in skin or muscles. In certain specific regions, the ratio of sensory to motor nerves can even reach 9:1 [25,26]. In terms of receptor types, researchers have found that fascia primarily contains multimodal free nerve endings. They can transmit mechanical, chemical, and noxious stimuli and form the most critical peripheral basis for pain perception [27,28]. Additionally, there are mechanoreceptors like Pacinian corpuscles, Ruffini corpuscles, and Golgi tendon organs, which are responsible for detecting subtle mechanical stimulation such as vibration, stretching, and light touch [29]. It has also been demonstrated that proprioceptors and muscle spindles are not merely attached to muscle fibers. Instead, they are mainly embedded within the epimysium. Because the epimysium is continuous with the fascia, these spindles can perceive multidirectional fascial tension. This finding suggests that fascia has the ability to actively modulate proprioceptive input [30]. Different types of fascia also show variations in their sensory functions. Take the thoracolumbar fascia as an example: it has a dense neural distribution, so its sensory function is especially important. In contrast, structures like the epimysium have relatively sparse innervation and mainly take part in local coordination [31,32]. Superficial fascia contains abundant autonomic nerve fibers. Its sensory function is closely related to autonomic activities such as vasoconstriction, vasodilation, and thermoregulation [33]. It may also play a role in regulating tension related to emotions and stress [32,34,35], further highlighting the functional differences among various types of fascia.

Because of this particular structure, fascia is able to perform multiple sensory and signal-transduction functions. First, it serves as a key mediator of proprioception and motor coordination. By transmitting the tension generated during muscle contraction, fascia provides continuous feedback to the central nervous system about posture and movement [27]. Fascia is also closely interwoven with nociceptive nerve fibers and is sensitive to inflammatory mediators. As a result, it becomes a significant source of musculoskeletal pain and participates in the modulation of pain signals [36]. Furthermore, fascia is connected to emotional regulation. Once sensory input has been processed by the central nervous system, it generates perceptions that incorporate both emotional and motivational factors. This offers a fascial-level explanation for psychosomatic disorders [25].

Some cutting-edge research has proposed innovative ideas. For example, according to the holographic fascia theory, cells communicate via electromagnetic fields or biophotonic signals, which endow fascia with memory and resonance capabilities [23]. Another study has suggested that proton hopping transport along the collagen and water molecule networks of fascia might occur faster than nerve conduction, thereby constituting a higher-speed signal transmission system [37]. These perspectives are still at the research stage, and their significance and reliability still need further validation. Furthermore, although the notion that “the fascia is the largest sensory organ” highlights its high neural density [38], the relative contribution and distribution of fascia in sensory integration compared to the skin and muscles still need to be clarified through more detailed functional studies.

### 2.3. Cell and Matrix Remodeling Functions

The fascia contains diverse, highly heterogeneous, and functionally specialized cell populations. Together with the ECM, these cells form a dynamic system and serve as the driving force behind remodeling processes [2]. At the cellular level, fibroblasts play a key role in the synthesis and maintenance of the ECM. They are highly sensitive to mechanical loading, and mechanical stimulation can promote the synthesis of more collagen [39]. Under the influence of chronic stress or injury, fibroblasts differentiate into myofibroblasts and participate in tissue remodeling and fibrosis through α-SMA-mediated contraction [40]. Single-cell studies further indicate that fibroblasts are not a homogeneous population but are composed of multiple subtypes, such as pro-inflammatory, fibrotic, and mesenchymal, and their synergistic imbalance is a key basis for pathological remodeling [41,42]. Additionally, fascia cells distributed at the edges of the myofascia are responsible for maintaining HA synthesis and interlayer lubrication [43]. Various resident immune cells are responsible for regulating the balance of inflammation and remodeling [44,45]. The newly discovered telocytes are involved in fascia repair and regeneration, providing a new cellular basis for self-repair after injury [46].

At the matrix level, the ECM constitutes the material basis for the mechanical and signaling regulation of fascia. Collagen determines its mechanical strength, with type I and type III collagens mainly providing tensile strength and showing a positive correlation with mechanical load, while the widespread distribution of type VI collagen suggests that fascia actively participates in mechanotransduction [47]. HA, as a key matrix component, regulates tissue states through molecular weight-dependent signaling: high molecular weight HA promotes tissue stability, whereas low molecular weight HA fragments drive inflammation and remodeling [21]. Mechanistically, mechanical stress is transduced through key pathways such as the Yes-associated protein, driving cellular phenotypes toward synthetic or contractile states and regulating ECM-related gene expression, thereby converting mechanical signals into transcriptional programs [48].

Based on the above mechanism, fascia exhibits significant tissue adaptability and repair capability. Traditional views hold that wound ECM mainly relies on local fibroblast migration and differentiation [49], but new evidence suggests that fascia can act as a matrix reservoir, carrying ECM tissue fragments for collective migration to participate in wound repair, which is more efficient than the single-cell mode [50,51]. This process works synergistically with the coagulation response to form a temporary matrix that promotes wound closure [52,53]. At the same time, progenitor cells within the fascia can participate in the orderly regulation of inflammation, proliferation, and remodeling stages according to spatiotemporal programming, thus ensuring orderly repair [54]. In summary, the remodeling function of fascia is essentially a dynamic, adaptive process driven by multiple cell subpopulations, carried by the ECM, and coordinated through mechanical transduction, enabling a continuous response to microenvironmental changes.

### 2.4. Metabolic, Immune, and Chemical Regulatory Functions

Fascia also acts as an active regulatory center, participating in local metabolism, immune responses, and the integration of systemic chemical signals. In terms of chemical sensing, receptors for various hormones and neurotransmitters are expressed by fascial cells. Being equipped with these receptors, the cells can respond to systemic endocrine signals with high sensitivity. Estrogen and relaxin can inhibit fibrosis by regulating fibroblast activity. They also influence ECM remodeling, thereby affecting fascial stiffness [55]. Meanwhile, the endocannabinoid system is widely distributed inside the fascia. It also participates in anti-inflammatory effects and pain modulation [56]. The function of fascia is also directly influenced by the local metabolic environment. When the environment becomes acidic, the contractility of myofibroblasts is enhanced. This enhancement leads to immediate stiffness. Lactic acid, however, produces a biphasic effect. In the short term, it causes pain, but over the long run, it promotes tissue repair [57]. This suggests that the mechanical state of the fascia is regulated by both systemic hormones and local metabolic signals.

As for immune regulation, the fascia forms a local immune microenvironment with its own responsive capabilities, where various immune cells such as mast cells, macrophages, and lymphocytes reside, and establishes a functional coordination mechanism with fibroblasts [39,44]. Under mechanical stress or infectious stimulation, the fascia can undergo adaptive reprogramming to mobilize specific immune cell populations and reshape the inflammatory response [58]. In pathological processes such as fibrosis, certain macrophage subsets significantly activate fibroblasts through the transforming growth factor-β signaling pathway and may even directly participate in the formation of ECM, suggesting that the immune system is closely linked to matrix remodeling and that both together promote pathological processes [42]. In the case of infection, the fascia also acts as a local defensive barrier and participates in the initial immune response [59].

Regarding fluid and substance exchange, fascia serves as a key interface connecting interstitial fluid, blood vessels, and the lymphatic system. Recent studies have confirmed that continuous interstitial fluid flow exists within fascial spaces [60,61]. The fascia contains a high amount of glycosaminoglycans. These molecules can bind large quantities of water, thereby influencing tissue water retention and the formation of edema [5,37]. Other findings suggest that the interstitial fluid inside the fascia does more than just transport nutrients and metabolic waste. Through fluid shear stress, it can directly modulate cellular behavior and immune responses as well. This helps explain why localized stasis of interstitial fluid quickly affects fascial function and pain perception [23]. Moreover, the superficial fascia contains a dense lymphatic network. When this network is mechanically coupled with tissue tension, it gives the fascia a function similar to that of a mechanical pump, which can actively promote lymphatic return [17,62].

Building upon these multilayered mechanisms, the “mechanical-metabolic model” proposes that the fascia is a bidirectional regulatory system. In other words, mechanical stress can influence HA metabolism and inflammatory states; conversely, metabolic changes can alter the mechanical properties of fascia. This creates a dynamic feedback loop [63]. Consequently, through endocrine sensing, immune modulation, and fluid exchange mechanisms, the fascia comprehensively regulates the local microenvironment and overall physiological state.

### 2.5. Neurovascular Integration of Fascia and Its Role in the Circulatory System

Fascia and the neurovascular system are closely interconnected both structurally and functionally. This close linkage allows them to work together in coordinating sensory transmission, circulatory regulation, and autonomic nervous system responses. Nerves and blood vessels are widely co-localized within the fascial system. In the joint-muscle-fascia complex model, neurovascular bundles are seen as core structures. These bundles run through all fascial layers and serve two purposes: supplying nutrients and transmitting information [64]. This feature is especially prominent in the superficial fascia. Nerves often run parallel to blood vessels and adipose tissue, forming stable neurovascular units [22]. About one-third of these units are made up of sympathetic nerve fibers. This suggests that these units are responsible not only for sensory input but also for regulating vascular dilation and constriction [65]. Concurrently, the fascia possesses a rich vascular network comprising arteries, veins, capillaries, and the lymphatic system. Through arteriovenous anastomoses, it ensures proper distribution of blood flow [33]. So, the function of fascia can extend to the level of systemic circulatory regulation. Receptors of the renin-angiotensin system, such as angiotensin II, are expressed by the fascia. This allows fascia not only to take part in regulating the local microenvironment but also to intervene in the systemic cardiovascular regulatory network. It does so by influencing vascular tone, inflammatory responses, and fibrotic processes, thus forming a key link between hemodynamics and connective tissue remodeling [66]. Other studies have shown that fascia helps maintain blood pressure by regulating peripheral vascular resistance. Under stressful conditions, it also promotes the redistribution of blood flow toward vital organs [67]. These findings indicate that fascia has specific abilities to become involved in circulatory regulation.

There is a mutual regulatory relationship between fascia and the autonomic nervous system. On one hand, mechanical stimulation or pathological changes in the fascia can trigger autonomic responses, such as sweating, changes in blood flow, or changes in body temperature [16]. On the other hand, sympathetic nerve activity can affect both pain and the functional state of the fascia. It does this through mechanisms such as regulating vascular tone and sympathetic-sensory coupling [32]. Furthermore, the remodeling of the sympathetic nervous system and the significant elevation of norepinephrine at myofascial trigger points confirm that pathological fascial states are closely linked to autonomic nervous system imbalance [68]. When fascial tension is elevated, it can mechanically compress nerves and blood vessels. This leads to local ischemia and metabolic disturbances. That mechanism provides a scientific explanation for functional disorders like chronic pain and fatigue [69].

Clinically, the condition of the fascia is a key mediator of neural function. Because nerves run through the fascia, their sensitivity to mechanical compression and to therapeutic interventions depends on their course within it [13]. Also, during surgical procedures, the relationship between vascular penetration points and fascial layers serves as an important basis for anatomical landmarks and for choosing surgical approaches [70]. Although the theory that fascia participates in regulating systemic circulation has theoretical merit, its systemic effects still require further quantitative evidence.

### 2.6. Fascial Embryology and Whole-Body Integrative Function

From the perspectives of origin and evolution, fascia should not be viewed as static, accessory connective tissue in adulthood, but rather as a system composed of dynamically formed, interconnected structures. Around the 6th week, the overall structure begins to form; from the 7th to the 8th week, cellular and collagen differentiation occur; and from the 9th week onward, there is a gradual transition to the tissue maturation phase. During this process, the epimysium and the fascial primordia exhibit a continuous homological relationship, indicating that the fascial and muscular systems do not represent a traditional “wrapper-and-wrapped” relationship, but rather share a common ontogenetic foundation. At the same time, autonomous movement can already be observed in the early stages of embryonic development. Under the influence of mechanical tension, mesenchymal tissue is remodeled into a fibrous network, thereby stimulating fibroblast differentiation and collagen deposition. This indicates that fascia is a prototypical structure of mechanically driven morphogenesis [17,71]. The periodic contractions of fibroblasts are synchronized with systemic rhythms such as respiration and the heartbeat, and influence fluid dynamics within the matrix. Consequently, the fascia serves as an integrative interface linking local mechanical events to systemic rhythms [37].

From an evolutionary perspective, fascia exhibits pleomorphic characteristics. In addition to its mesodermally derived mesenchymal origin, portions of the cranio-cervical fascia also originate from the neural tube of the ectoderm [24]. This dual origin provides the anatomical basis for fascia’s ability to provide both mechanical support and neural regulation, while also explaining the close congenital connection between fascia and the nervous system. Fascia exhibits continuous homology with the extracellular matrix and mesodermal skeleton at different stages of biological development, suggesting that fascia is a fundamental support system that has been preserved throughout the evolution of multicellular organisms from simple structures to complex organ systems [23,72]. In terms of evolutionary sequence, fascia and cerebrospinal fluid are among the first extensive networks to form, providing a scaffold for the subsequent differentiation and growth of other bodily systems and structures. At the level of tissue development, fascia participates in the construction of embryonic “compartmentalization.” During differentiation, the mesodermal stroma forms a multilayered membrane-like structure. The outer layer develops into the intrinsic fascia of organs, the middle layer forms functional fascial spaces containing adipose tissue, and the inner layer differentiates into serosal structures [73,74].

The plasticity of fascia persists throughout life, and its structure is regulated by aging, mechanical loading, and pathological conditions. Aging promotes collagen cross-linking and reduces the tissue’s adaptability. When mechanical loading is lacking, the water-retention capacity and glide properties of fascia are reduced. Moderate mechanical stimulation, on the other hand, promotes cellular and matrix remodeling. Through this promotion, both structural and functional stability are maintained [1]. Such dynamic adaptability further serves to highlight the mechanical properties of fascia.

**Figure 1:**
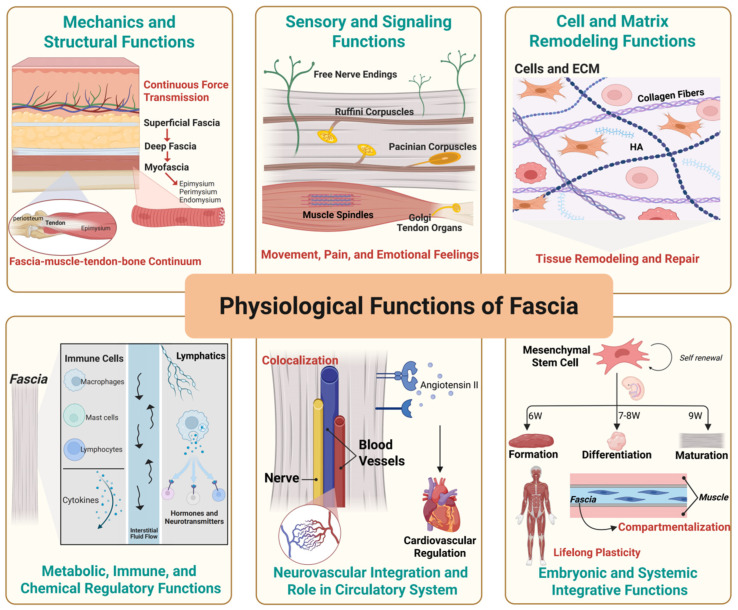
Schematic Diagram of the Six Major Physiological Functions of Fascia and Their Key Components. Mechanical and Structural Functions: Demonstrates the continuous force transmission system extending from superficial fascia, deep fascia, and myofascia to the tendon, periosteum, and bone, highlighting the fascia–muscle–tendon–bone continuum. Sensory and Signaling Functions: Illustrates the layered distribution of sensory receptors and neural structures, emphasizing the contribution of fascia to sensory modulation and nociceptive processing. Cellular and Matrix Remodeling Functions: Emphasizes the interactions between cells and the ECM, including fibroblasts, myofibroblasts, collagen fibers, and HA. Metabolic, Immune, and Chemical Regulatory Functions: Demonstrates the presence of abundant immune cells, cytokines, hormones, and neurotransmitters within the fascia. Neurovascular Integration and Circulatory Regulation: Illustrates the joint participation of neurovascular units in the regulation of local and systemic cardiovascular circulation. Embryonic and Systemic Integration Functions: Presents the formation, differentiation, and maturation of the fascia along a timeline, highlighting its lifelong plasticity, compartmentalization, and systemic integrative role. Created in https://BioRender.com.

To summarize, the function of fascia rests on two things: its continuity during embryonic development and the processes of mechanical remodeling. Because of this basis, fascia becomes a fundamental structure that links mechanical stimulation, developmental programs, and systemic physiological regulation (Figure 1).

## 3. Fascial Dysfunction and Common Pathological Conditions

**Figure 2:**
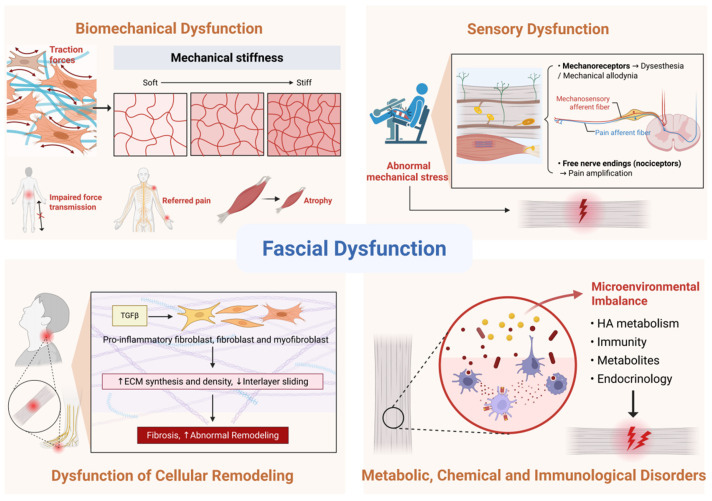
Schematic Diagram of Multidimensional Pathological Mechanisms of Fascial Dysfunction. Biomechanical dysfunction: Physicochemical changes in the matrix lead to bidirectional alterations in fascial stiffness (stiffening or softening), resulting in impaired force transmission, referred pain, and muscle atrophy, ultimately causing pain and movement disorders. Sensory dysfunction: Abnormal fascial mechanical stress disrupts the normal activation patterns of sensory receptors, leading to nociceptor-driven pain amplification and mechanoreceptor-mediated dysesthesia or mechanical allodynia. Cellular remodeling dysfunction: Signals such as TGF-β drive the transformation of fibroblasts into myofibroblasts and activate pro-inflammatory fibroblast subpopulations, leading to increased ECM synthesis, elevated matrix density, and reduced interlaminar gliding, ultimately resulting in fibrosis and abnormal tissue remodeling. Metabolic, chemical, and immune dysregulation: Abnormal HA metabolism, dysregulation of immune cell function, accumulation of metabolic byproducts, and endocrine factors collectively lead to microenvironmental imbalance. These microenvironmental changes interact causally with the aforementioned dysfunctions, jointly maintaining chronic pain and motor dysfunction. Created in https://BioRender.com.

Fascial dysfunction has increasingly been recognized as an important contributor to various musculoskeletal pain conditions and movement disorders. In many cases, fascial alterations emerge as part of a complex pathological cascade involving mechanical overload, inflammation, metabolic imbalance, and impaired tissue repair. Once established, these alterations may further contribute to disease progression by affecting multiple functional domains (Figure 2).

### 3.1. Biomechanical Dysfunction

Among the multiple consequences of fascial dysfunction, biomechanical alterations represent one of the most extensively studied aspects. Fascial mechanical abnormalities may develop in response to pathological loading, inflammation, aging, or impaired tissue repair. During this process, physicochemical changes take place in the matrix, which usually show up as increased matrix density. Why does this happen? Alterations in HA metabolism and increased collagen cross-linking are the reasons. These two changes lead to higher matrix viscosity and a reduced ability to glide between layers [4]. Imaging studies have confirmed this: patients with chronic pain typically present with fascial stiffness and poor gliding capacity [75]. Eccentric stress testing provides further evidence. Damage to the deep fascia results in more edema and stiffness, and this finding correlates positively with the intensity of clinical pain [76]. At the microscopic level, these changes come from an imbalance among fibroblast subpopulations inside the fascia. Some pro-fibrotic and inflammatory subpopulations proliferate, which then leads to abnormal remodeling of the ECM [58]. Accordingly, restoring fascial gliding capacity has been shown to significantly alleviate pain. These findings support the view that impaired fascial gliding capacity may represent an important mechanical contributor to persistent pain [77,78].

All the alterations described above cause abnormalities in the overall mechanical properties, and these abnormalities go in both directions. For example, they may manifest as pathological stiffening, such as the increased stiffness of the cervical fascia seen with forward head posture [79]. Or as softening, for instance the reduced load-bearing capacity in plantar fasciitis caused by degeneration of the collagen network [80]. However, in some chronic pain cases, fascial thickening and increased stiffness do not happen at the same time. Thickening is usually attributed to repeated microtrauma or cumulative fibrosis. But when stiffness does not increase, that may relate to changes in collagen tissue structure or matrix degradation [81]. So the pathological process is not just about hardening; it is really a complex remodeling process. Abnormal mechanical states trigger clinical symptoms mainly through two mechanisms. First, densely distributed nociceptors within the fascia are directly activated, and that triggers pain [4]. Second, disturbances in force transmission occur at both local and systemic levels. Because the fascia is continuous, local abnormal tension propagates along the network. This may cause pain that radiates to extensive areas [69]. If force transmission between the fascia and muscles becomes restricted, adaptability and muscle growth are impaired, and, in some cases, this may even lead to muscle atrophy [8].

Pathological mechanical changes in the fascia are not uniform; they vary. And functional symptoms differ depending on the type of fascia and the specific pathological condition. Take plantar fasciitis as an example: you can observe the “thickest is weakest” phenomenon there, which reflects the spatial heterogeneity of the lesion [82]. Moreover, phenomena like softening or thickening without hardening indicate that using only morphological indicators makes it hard to accurately describe the functional state of the fascia. So we need a comprehensive assessment that combines mechanical and functional parameters [80,81]. Thus, fascial mechanical dysfunction unfolds as a continuous process: it starts with matrix abnormalities, then progresses to changes in tissue mechanics, and finally leads to neural activation and imbalances in force transmission. It acts both as a consequence of underlying pathology and as a contributor to persistent symptoms.

### 3.2. Sensory Dysfunction

Fascia is an important sensory organ. When it is placed under abnormal mechanical stress, the activation patterns of its receptors become disrupted. This disruption then gives rise to sensory input that is both abnormal and harmful. Evidence from experiments suggests that fascia alone may act as an independent source of pain. For example, when hypertonic saline is injected into fascia, the pain responses it produces are stronger and last longer than those induced in muscle. This finding points to a significant capacity within the fascia to generate harmful input signals [83,84]. What is the neurological basis behind this phenomenon? It lies in the reorganization of the nociceptive system. This reorganization includes two things: excessive proliferation of sensory nerve fibers and overinnervation by the sympathetic nervous system [45]. The sympathetic nervous system lowers receptor thresholds by releasing norepinephrine. In doing so, it forms an amplifying loop, which is known as “sympathetic-sensory coupling” [68]. At the same time, inflammatory mediators and metabolic byproducts keep accumulating. These substances continuously sensitize receptors, and through this process, peripheral pain impulses are sustained [85].

Viewed from this perspective, mechanical abnormalities act as significant amplifying factors. In vitro studies have shown the following: when matrix stiffness increases, it directly boosts the release of neuronal action potentials, raises synaptic density, and enhances Schwann cell function. Consequently, nerve endings become sensitized, their activation thresholds are lowered, and noxious stimulation is amplified [86]. If peripheral stimulation persists, central sensitization is induced. This condition then increases pain processing in the spinal cord and higher-level centers. Through this sequence, a local injury is transformed first into local pain, and that local pain may ultimately progress into systemic pain [4]. Additionally, these abnormal mechanical signals interfere with normal proprioceptive input, causing distortion of sensory information transmitted to the brain. This affects cortical representation and motor control, thereby exacerbating functional impairment [8]. Furthermore, shared innervation among fascia, muscles, and skin leads to the convergence of sensory inputs and the amplification of pain. Their relative contributions to pain remain a subject of debate; fascial pain is often misinterpreted by the central nervous system as originating from muscles or skin, resulting in referred or diffuse pain [28]. Some scholars also suggest that abnormalities in interstitial fluid flow and signal transmission within the fascia may be misinterpreted by the central nervous system as pain signals [23]. This hypothesis offers an explanation for chronic pain cases where imaging studies reveal no obvious structural abnormalities, but further in-depth research is still needed.

### 3.3. Dysfunction of Cellular Remodeling

Dysfunction of fascial cellular remodeling is an internal factor that leads to fibrosis, muscle contractures, and chronic wounds that are difficult to heal. Essentially, this is caused by an imbalance in the processes of cellular regeneration, differentiation, and repair, which in turn triggers tissue imbalance and ultimately results in persistent pathological remodeling. At the cellular level, the core mechanism is dysregulation of the fibroblast system. When abnormal loading or inflammatory stimuli are applied, pathological reprogramming can be induced. As a result, collagen-related genes become overexpressed in these cells, and the cells themselves are transformed into effector cells that secrete excessive ECM [87]. This process is not just about simple cell proliferation; instead, it involves the widespread activation of multiple subpopulations. That is why when interventions are designed to target single molecules, their efficacy tends to be limited [42]. At the spatial organizational level, the structural arrangement of fibroblasts becomes abnormal. Under normal regenerative conditions, fibroblasts establish an interconnected network through remodeling of their intracellular actin cytoskeleton, thereby enabling dispersed and coordinated contraction. However, under the influence of signals such as transforming growth factor-β1 (TGF-β1), α-SMA binds to actin, forming a concentrated and irreversible contraction pattern that leads to localized stress concentration and structural stiffening [40,88]. Spatial biology studies have further demonstrated that fibroblasts within deep fascia can form specific tissue units; these units tend to solidify into aggregate patterns, which, as fibrosis progresses, promote fibrosis and thereby exacerbate the pathological trends of tissue remodeling [89].

During the repair process, cellular origin-related or substance transport-related abnormalities further exacerbate pathological evolution. Spatiotemporal dysregulation of precursor cell differentiation within the fascia may lead to an insufficient supply of repair cells [54]. At the same time, the fascia itself functions as a reservoir and transport vehicle for the ECM. Dysfunction of the fascia limits the effective delivery of repair substances to the injury site; Key mechanisms of this process include the migration of En1-positive fibroblasts and abnormalities in cell adhesion [9]. Studies on scarless healing in fetal skin indicate that successful repair depends not only on fibroblasts failing to differentiate into myogenic fibroblasts but also on their ability to form appropriate spatial actin networks, thereby achieving a coordinated distribution of tension [88]. It can thus be concluded that the key to fascial remodeling disorders lies not only in increased ECM deposition but also in the pathological fixation of the fibroblast lineage and alterations in its spatial organization. This process transforms the fascia from a plastic tissue into a structurally rigid, fibrotic state, ultimately leading to functional changes.

### 3.4. Metabolic, Chemical, and Immunological Disorders

Imbalances in the metabolic, chemical, and immunological microenvironment of the fascia are key underlying mechanisms that trigger mechanical abnormalities and pain. Several studies have shown that inflammation and edema are already present in the fascial environment before obvious structural changes become apparent; this is a significant characteristic of early pathological processes in the microenvironment [90]. Metabolic disturbances in HA represent an early manifestation. In conditions of restricted movement, aging, or metabolic dysfunction, these molecules aggregate to form high-viscosity networks, thereby altering the rheological properties of the local matrix and restricting interstitial fluid exchange and substance diffusion [91]. However, given that the rheological properties of HA are regulated by movement, pH value, and temperature, and have a certain reversibility, it provides an important therapeutic entry point for early intervention [40].

Imbalances in the fascial immune microenvironment and chronic inflammation are factors that promote pathological remodeling. In superficial fascia, mast cells can release mediators such as histamine and prostaglandins, thereby exacerbating inflammation and hyperalgesia [44]. Similarly, in deep fascia, the differentiation of T-cell subsets and the polarization of macrophages are both imbalanced; these cells, together with fibroblasts, form a regulatory network governing the “inflammation-fibrosis” axis through signaling pathways such as tumor necrosis factor [4]. Here, macrophages can modulate fibroblast proliferation and collagen synthesis via paracrine mechanisms, directly driving ECM remodeling and the fibrotic process [92]. Aging, chronic disease, and malnutrition compromise immune homeostasis and promote the spread of inflammation along the fascial surface [59].

Alterations in the local metabolic and chemical microenvironment form the basis for persistent pain. In areas affected by chronic disease, increased concentrations of serotonin, glutamate, and lactate are frequently observed [93]. Local acidification not only directly activates nociceptors but also exacerbates myofibroblast contraction, leading to increased tension and intensified pain. Furthermore, lactate shifts from a short-term repair signal to a fibrogenic factor, promoting ECM deposition and vascular remodeling [57]. These microenvironmental changes are often accompanied by microcirculatory instability, manifested as abnormal blood flow fluctuations and tissue hypoxia, which further impede the clearance of metabolic byproducts [94]. Moreover, fluctuations in body temperature and altered heat sensitivity observed during both the acute and chronic phases of fascial inflammation similarly reflect disturbances in metabolic balance [95,96].

At the systemic level, endocrine and metabolic factors participate in systemic remodeling by modulating the fascial microenvironment. Given that fascial cells express estrogen and relaxin receptors and are sensitive to these hormones, this may partially explain the high prevalence of myofascial pain in women [55]. Chronic hyperglycemia leads to collagen stiffening at the nanoscale through glycation and oxidative processes, while simultaneously disrupting the immunoregulation of fibroblasts, thereby exacerbating inflammation and impairing wound healing, and constituting a major basis for diabetic complications [97,98]. Furthermore, angiotensin II receptors are overexpressed by myofascial fibroblasts, making these cells targets for signaling from circulating hormones. This finding points to a link between fascial health and cardiovascular status. It also helps us understand why fascial mechanical abnormalities are often observed in conditions like hypertension [99]. Overall, metabolic, chemical, and immunological dysfunctions in fascia are interconnected. These processes mutually reinforce each other and contribute to persistent pathological remodeling.

## 4. Assessment and Diagnosis of Fascial Function: Testing Techniques and Instruments

In recent years, this field has seen rapid advances in fascial research. These advances have not only deepened our understanding of how fascia functions but have also pushed forward significant progress in research methods and assessment techniques. After looking closely at recent studies, one can see a clear trend: the field is gradually moving away from empirical descriptions and toward quantitative, standardized measurements. A multidimensional assessment system is now being built step by step. This system focuses on the mechanical, structural, and biological characteristics of fascia, as well as its mechanisms of action. With such a system, the objective diagnosis of fascial dysfunction and the evaluation of treatment efficacy become possible.

### 4.1. Clinical Palpation and Imaging Assessment

In clinical practice, palpation remains the most basic method for assessing fascia. It can be used to find tender points, detect fibrotic changes, and identify pain referral patterns. Because of this, it serves as an effective screening tool for fascial pain syndromes. Studies have shown that, in areas of fascial hardening, the degree of tenderness correlates with pain intensity. It may also contribute to certain pain referral patterns [84,100]. That said, palpation mainly reflects the movement of skin and superficial tissues. When it comes to assessing the displacement and mechanical behavior of deep fascia, this method lacks consistency. It also struggles to reliably map the condition of deep fascial structures [101]. To improve the objectivity of assessment, digital palpation techniques represented by MyotonPRO have emerged in recent years. This device enables non-invasive measurement of tissue stiffness, elasticity, and viscoelastic properties, providing quantitative parameters that reflect the mechanical status of soft tissues. Existing studies have applied MyotonPRO to the evaluation of fascia-related chronic pain and musculoskeletal dysfunction, demonstrating good reliability and promising clinical applicability. Therefore, MyotonPRO may serve as an important complementary tool bridging traditional palpation and imaging techniques, offering quantitative evidence for the objective assessment of fascial functional status [102,103]. Importantly, MyotonPRO measurements require a strictly standardized protocol, including probe placement, body posture, muscle condition, and testing environment. Without such standardization, parameters such as fascia tone and stiffness may show reduced validity and limited comparability across anatomical regions and subjects [104].

Ultrasound has become an indispensable tool for evaluating fascial structures. In fact, it gives a clear view of linear and hyperechoic fascial structures. It can be used to measure their thickness and to spot abnormalities like thickening, hypoechoic areas, and blurred margins [105,106,107]. Dynamic ultrasound also allows functional assessment: by quantifying the sliding motion between subcutaneous tissue and muscle, it reflects how severe adhesions and densification are [108,109]. Recent studies have demonstrated high reliability (intraclass correlation coefficient (ICC) > 0.89) for ultrasonographic measurements of plantar fascia thickness and echogenicity [110]. A critical methodological challenge is the anisotropy effect: deep fascia exhibits highly organized, parallel-aligned collagen fibers, and even minimal changes in probe angulation (as little as ±5°) can cause significant variations in both echogenicity and thickness measurements [111]. Therefore, standardized protocols are imperative. Operators must maintain the ultrasound beam perpendicular to the fascial plane and control for transducer pressure, patient posture, imaging planes, and probe positioning to ensure reproducible measurements [112].

Magnetic resonance imaging (MRI) provides even more detailed structural information. It can assess where thickening, tears, and edema are distributed [90,113]. T2 mapping techniques can quantitatively detect fascial edema [76], while diffusion tensor imaging is used to analyze how the fascia deforms and transmits force [114]. Studies have demonstrated that MRI-based plantar fascia thickness measurements show excellent repeatability as high ICCs (r = 0.988–0.990) [115]. Major limitations include relatively limited spatial resolution for thin fascial layers, high cost, and limited accessibility. Also, it remains affected by scanner-specific factors, sequence parameters, and post-processing methods, which may limit cross-center comparability. Furthermore, MRI primarily provides static structural information and is less suitable for evaluating dynamic fascial behavior. Computed tomography (CT) is mainly used to assess general morphological changes in the fascia and subcutaneous tissue, such as those caused by lymphedema [116]. The reliability of CT-based fascial assessment has been documented in multiple studies. Analysis of abdominal fascial layers demonstrated excellent interobserver reliability, with ICC values ranging from 0.991 to 0.995 [117]. Standardized CT assessment requires consistent acquisition parameters, including slice thickness, reconstruction algorithms, field of view, and contrast-enhancement protocols [118].

Shear-wave elastography (SWE) is currently the gold standard technique for assessing fascial stiffness. This technique calculates the elastic modulus of tissue based on shear wave velocity, thereby enabling quantitative assessment and delivering reproducible measurement results [119]. Studies have shown that SWE consistently reflects changes in fascial stiffness across various pathological conditions, and its results correlate with symptom severity. For example, thoracolumbar fascial stiffness increases in chronic low back pain [120], abnormal localized stiffness is observed in plantar fasciitis [82], and fascia stiffness changes in neck pain, all of which are independent of muscle mass [79]. Furthermore, SWE can be used to assess the efficiency of force transmission between fasciae. By detecting changes in stiffness of adjacent fascia under tensile stimulation, the efficiency of force transmission within fascial chains can be quantified [18]. In contrast, elastic deformation images depend on the pressure applied by the operator; therefore, they are only semi-quantitative in nature and are increasingly being replaced by SWE [80]. Regarding reliability, ultrasound measurement of fascial thickness and stiffness has demonstrated good to excellent reliability, with intraclass correlation coefficients (ICC ≥ 0.80) [81]. To optimize reproducibility, it is recommended that SWE measurements be performed by experienced ultrasonographers, targeting superficial body regions, and with the tissue positioned beyond its slack length [119].

In recent years, various techniques have been developed to enable more dynamic and real-time functional assessments. Ultrasound palpation combines imaging diagnostics with physical examination techniques. By applying pressure via a probe, this technique allows for the intuitive localization of relevant fascial layers while also eliciting pain responses, thereby improving the accuracy of lesion localization [121]. In the evaluation of acute fascial compartment syndrome, non-invasive ultrasonic elasticity measurement also offers a new method for estimating pressure within the fascial compartment [122].

Although palpation has its subjective aspects and limitations, it must be emphasized that it remains of inestimable value in clinical practice for the localization and diagnosis of pathological conditions. However, future research is needed to determine how to standardize and quantify palpation. Furthermore, changes in fascial stiffness are pathologically specific and cannot be generalized. Given that morphological abnormalities do not necessarily lead to functional impairment, functional testing and clinical symptoms must be integrated to assess whether the structural changes observed on imaging are the true cause of pain.

### 4.2. Tissue and Materials Mechanics Assessment

Unlike clinical examinations and imaging studies that focus on functionality, tissue and material mechanics focus on the mechanical properties and basic structures of the fascia itself and translate clinical descriptions such as “stiff” or “loose” into measurable and reproducible parameters.

Laboratory mechanical testing is the primary method for analyzing the material properties of fascia. Such experiments are usually conducted on in vitro tissue or technical models. Through standardized tensile, compressive, or shear tests, parameters like elastic modulus, tensile strength, and elongation at break can be determined. They can reveal the load-bearing capacity and structural integrity of fascia [123]. On this basis, strain and stress measurement systems allow real-time recording of fascial deformation and stress distribution during loading. By doing so, quantitative analysis of force transmission relationships and anisotropic properties becomes possible [18]. Meanwhile, rheological methods focus on the viscoelastic properties of fascial tissue, such as stress relaxation, hysteresis, and nonlinear behavior. They can also be used to simulate the rheological behavior of HA under various shear conditions [86]. At the microscopic level, atomic force microscopy (AFM) enables measurement of local fascial elasticity with nanometer-scale resolution. As a result, pathological stiffening of the fascia in diabetic patients has been confirmed at the nanoscale, which points to a direct link between metabolic abnormalities and micromechanical changes [97]. Furthermore, specialized devices such as sliding resistance tests can quantify changes in friction between fascial layers. These tests are also employed to assess the mechanical effects of fluid separation or manual interventions, thus providing direct evidence of therapeutic mechanisms [78].

Compared to laboratory methods, clinical settings prioritize accessibility and reproducibility. This priority has driven the development of numerous portable devices for mechanical assessment. Injection pressure monitoring, for example, uses a puncture needle equipped with a fiber-optic pressure sensor. By tracking pressure changes at the needle tip, the characteristics of the fascia layer being punctured can be identified. This not only improves the precision of nerve blocks but also allows quantitative assessment of fascial density [19,124]. Indentometers calculate tissue stiffness based on standardized penetration depth and resistance, making them a simple method for clinical screening and for monitoring treatment outcomes [125]. Similarly, devices like FascialSense or PACTSense, which rely on fiber-optic or pressure sensor technology, can simulate palpation to generate quantitative stiffness values. The results obtained from these devices show good consistency with the MyotonPRO, a widely recognized and commonly used digital palpation system, and are relatively reliable in terms of validity and reliability [126,127]. Notably, the use of these devices requires adherence to standardized measurement protocols. However, these portable devices do not reflect the properties of a single fascial layer but rather the mechanical response of a multi-layered tissue complex. Thus, their ability to perform layered analysis is limited.

The challenges and future prospects in the field of fascial tissue mechanics stem from the aforementioned contradiction between experimental and clinical practice. A trade-off always exists among cost, portability, and measurement accuracy. Laboratory equipment provides high precision and strong mechanistic insight, but it cannot be used in clinical or in vivo settings. On the other hand, portable devices are easy to deploy, yet they still face challenges in terms of depth resolution and targeting. Specimen preparation, sample dimensions, loading rates, preconditioning cycles, and environmental conditions vary widely across studies, making cross-study comparisons difficult. Therefore, future research needs to focus on developing an evaluation framework that can integrate different assessment criteria and effectively correlate the mechanical parameters of ex vivo materials with in vivo functional performance. And to enhance reproducibility, researchers should adopt established guidelines for soft tissue mechanical testing. Only then can the functional state and physical properties of fascia be accurately characterized.

### 4.3. Morphological and Microstructural Assessment

Assessing fascial morphology and microstructure involves several aspects: reconstructing the collagen network, detecting changes in cellular phenotype, and identifying abnormalities in neurovascular distribution. These assessments provide a structural basis for explaining pain, stiffness, and contractures at the microscopic level.

#### 4.3.1. Histology and Structural Staining

To characterize the cellular state of the fascia, immunolabeling techniques are often used. Among these markers, α-SMA helps assess the degree of myofibroblast activation. It also serves as a key indicator for evaluating fascial contractility and the progression of fibrosis [1]. With neuroimmunological multistain staining (S100, PGP9.5, tyrosine hydroxylase), semi-quantitative analysis of the fascial neural component becomes possible. This technique reveals significant differences in nerve density across different fascial regions. It also confirms that sympathetic nerves are stably distributed within the fascia [32]. Conventional staining methods can be used to examine the structural basis of the ECM. Masson staining reflects the state of collagen deposition and its organizational structure. It is commonly used to assess the degree of fibrosis and matrix remodeling [128]. When viewed under polarized light, Sirius Red can distinguish between Type I and Type III collagen. It also clearly delineates the interface between fascia and muscle, thereby helping to better understand their spatial relationship [30]. Importantly, the reliability and reproducibility of histological assessment depend heavily on standardized tissue processing protocols, including specimen harvesting, fixation, embedding, section orientation, staining procedures, antibody dilution, and image analysis methods [129]. Previous studies have demonstrated that variations in fixation-induced shrinkage and tissue dehydration can significantly affect measurements of fascial thickness and collagen organization [65]. This indicates inherent methodological limitations in histological assessment. Therefore, such findings should be treated with caution and interpreted together with other results.

#### 4.3.2. High-Resolution Microscopy and Structural Analysis Methods

The use of high-resolution microscopy and structural analysis methods allows for in-depth study of fascia, going beyond what is visible to the naked eye and elevating research to the microscopic level. Through three-dimensional microscopic images, the three-layered structure of deep fascia was confirmed spatially for the first time, and it was observed that the collagen fibers in the middle layer exhibit distinct local mechanical adaptability [130]. Using scanning electron microscopy, it is possible to observe the three-dimensional arrangement of collagen fibers within the fascia, their interface with adipocytes, and age-related changes in their ultrastructure at high resolution [131]. Through cryofixation and low-vacuum electron microscopy techniques, artifacts caused by contraction can be minimized. Based on this, studies have discovered that the fascia is in a pre-tensioned state under conditions approaching physiological tension for the first time, which constitutes the ultrastructural basis for its function [11]. By combining epoxy resin embedding and confocal microscopy, three-dimensional non-destructive reconstructions of the boundaries between nerves, blood vessels, and fascia can be performed. It provides an anatomical basis for understanding the functional zones of the fascia [13].

Building on this, multimodal multiphoton imaging techniques can simultaneously acquire structural and biochemical information. Second-harmonic imaging technology can quantitatively analyze the arrangement and supramolecular structure of collagen, while two-photon fluorescence imaging and fluorescence lifetime imaging are used to visualize changes in the microenvironment. Furthermore, the combination of AFM and multiphoton technology allows for the simultaneous assessment of mechanical properties and molecular modification states at the nanoscale, making it a cutting-edge technological combination for analyzing pathological changes in fascia [97]. Moreover, emerging bone ultrasound technology, combined with image post-processing, can extract and visualize fibrous connective tissue networks, observe fascial chains in vivo, and provide intuitive evidence to support hypotheses regarding their continuity [64]. Imagingomics has now been applied further to the detection of invisible structures. By using textural features such as gray-scale variations in MRI, abnormalities in collagen alignment and matrix heterogeneity can be detected. In this way, structural abnormalities can be identified at an early stage, even before conventional imaging reveals any visible changes. This approach is also used for risk prediction of pathological changes and for disease staging [132]. With optical coherence tomography (OCT) and its derivative technologies, multifaceted optical analysis capabilities are provided. Basic OCT studies have confirmed that fascia possesses a layered structure, high diffusivity, and limited light transmittance [133]. During the puncture process, needle-based OCT can acquire real-time images at the micrometer scale. Based on attenuation rates, it also allows quantitative analysis of tissue properties to be performed [134]. By measuring birefringence parameters, polarization-sensitive OCT can reflect the alignment of collagen. In doing so, it establishes an optical correlation between structural integrity and mechanical properties [135]. In addition, studies have demonstrated high diagnostic performance for OCT-based muscle-fascia differentiation, with reported sensitivity, specificity, and overall accuracy exceeding 0.89 [134].

Taken together, these high-resolution techniques have greatly enhanced our ability to analyze fascial microstructure. Nevertheless, many of them remain in the preclinical stage or are confined to specialized research fields. Their clinical application is limited by cost, operational complexity, and a lack of standardization. For example, quantitative parameters derived from second-harmonic generation imaging, confocal microscopy, or OCT may vary according to imaging depth, optical alignment, and segmentation methods [136,137,138]. To transform these technologies from mere cutting-edge innovations into widely available diagnostic tools in the future, it is essential to develop consensus guidelines for specimen preparation, imaging acquisition, and data analysis, establish reference standards and quality control materials, promote the simplification of techniques and the standardization of parameters, and clarify the relationship between quantitative indicators and disease staging and prognosis.

### 4.4. Assessment of the Metabolic and Biochemical Microenvironment

#### 4.4.1. Microcirculation and Blood Circulation

The assessment of microcirculation and blood circulation within the fascial layers reflects the functional state of the fascia as a metabolic exchange interface. Infrared thermal imaging and laser Doppler flowmetry are currently the most widely used non-invasive techniques for functional assessment. Medical infrared thermal imaging records heat radiated from the skin surface, thereby indirectly providing information on blood circulation and metabolism in the deep fascia [96]. In cases of fasciitis, localized hyperthermia is often observed during the acute phase, whereas hypothermia is more common during the chronic phase. At the same time, asymmetry in heat distribution between the left and right sides can serve as a characteristic indicator for rapid screening of abnormalities [95]. A reliability study of infrared image analysis for upper trapezius myofascial trigger points reported moderate to excellent intra-rater ICC values ranging from 0.591 to 0.993 and inter-rater ICC values ranging from 0.615 to 0.918, supporting the reproducibility of this technique under standardized acquisition conditions [139]. Furthermore, dynamic thermal imaging can be used to identify hotspots on the skin surface and indirectly assess perivascular distribution. However, it is difficult to precisely localize the fascial layer due to its limited penetration depth. Therefore, this technique is more suitable for preoperative screening [70]. Laser Doppler flowmetry provides a continuous quantitative assessment of microvascular perfusion by detecting frequency shift signals generated by the movement of blood cells. Studies have shown that microcirculatory fluctuations are significantly exacerbated in patients with chronic neck pain at rest, while a characteristic hyperemic response may occur following effective treatment. Therefore, this method is suitable not only for evaluating pathological conditions but also for monitoring treatment outcomes [140]. Doppler ultrasound, serving as an adjunctive tool, can be used for dynamic observation of local blood flow changes [82].

However, infrared thermal imaging is easily affected by environmental factors and operational conditions. Its reproducibility depends heavily on strict standardization. To ensure reproducible results, strict standardization is essential across several domains: environmental conditions, including ambient temperature control and acclimatization period; patient preparation, encompassing rest, appropriate clothing, and avoidance of vasoactive substances, as well as standardized posture; image acquisition parameters, such as camera distance and emissivity settings; and analysis methods, particularly region-of-interest selection. Laser Doppler only captures instantaneous local blood flow velocity, which makes it difficult to assess the overall microcirculatory status at the fascial level. Given these limitations, such functional indicators are better treated as supplementary information. They should always be interpreted together with clinical symptoms and imaging findings.

#### 4.4.2. Detection of Tissue Metabolism and Chemical Signaling Molecules

In vivo detection of fascial metabolism and chemical signaling molecules is key to revealing the fascia’s role as an active pathological structure. It also provides an important means of identifying the source of pain, quantifying the inflammatory burden, and dynamically monitoring treatment responses. Currently, in vivo microdialysis stands as the only technique that can directly sample fascial interstitial fluid and quantify multiple biochemical substances. For this reason, it is considered the gold standard for assessing the chemical microenvironment and metabolic status of fascia. By implanting a semipermeable catheter into the target fascial region and then perfusing and continuously collecting interstitial fluid at a constant rate, dynamic monitoring of the local biochemical environment becomes possible [141]. This technique has two core applications. The first is metabolite analysis: lactate, glucose, pyruvate, and other substances can be detected, providing a direct reflection of the tissue’s energy metabolism [85,94,142]. Second, it involves the analysis of pain and inflammatory mediators, enabling the quantification of a series of key biochemical signals, including inflammatory factors, neuropeptides, catecholamines, and pH levels [141,143]. What gives microdialysis its advantage is not just its ability to measure concentrations. It can also dynamically capture the post-stimulation biochemical response patterns in vivo, so as to observe functional changes. For reliable assessment, microdialysis requires rigorous methodological standardization, including accurate probe placement within the target fascial region, standardized perfusion flow rates, sufficient equilibration periods, and calibration of relative recovery [144]. However, Methodological investigations have demonstrated that inter-individual variability accounts for nearly half of the total variability in microdialysis measurements, whereas catheter-related variability contributes less than 20%. To allow routine clinical use of microdialysis, further research efforts should aim at understanding and minimizing method-related variability. Standardized protocols must include consistent catheter insertion depth and placement, controlled perfusion rate and sampling intervals, rigorous calibration procedures, appropriate catheter membrane specifications matched to target molecules, and standardized sample handling and analysis protocols [145].

To comprehensively analyze the metabolic and chemical state of the fascia, it is necessary to combine multiple complementary techniques. Microdialysis is often combined with laser Doppler flowmetry or the ethanol efflux method to achieve a comprehensive assessment of metabolism and blood flow, which helps distinguish between ischemic and neurogenic inflammatory mechanisms [94]. Phosphophosphorylated magnetic resonance spectroscopy provides a non-invasive assessment of deep tissue energy metabolism, allowing for the measurement of adenosine triphosphate, creatine phosphate, and tissue pH; it is an important tool for studying systemic fascial metabolic abnormalities such as fibromyalgia [85]. In addition, highly sensitive detection techniques such as pH microsensors and immunoaffinity capillary electrophoresis/electrochromatography can be used together with microdialysis. These methods allow precise quantification of local pH levels, as well as ultra-low concentrations of neuropeptides and catecholamines [143].

Taken together, what these technologies reveal is another important dimension of how the fascia perceives its environment: chemical sensitivity. Looking ahead, if microdialysis samples are integrated with multi-omics analysis, a comprehensive elucidation of the chemical signaling networks under both healthy and diseased conditions in the fascia could be achieved. New biomarkers and potential intervention targets might then be identified.

### 4.5. Assessment of Mechanisms at the Molecular and Cellular Levels

#### 4.5.1. Molecular Expression and Omics

Driven by advances in molecular expression and omics technologies, the way fascial function is assessed has shifted. No longer confined to the tissue level, it has moved down to the cellular and molecular levels. In doing so, it has become an indispensable tool for elucidating cellular activity, remodeling processes, and signaling pathways. Spatial omics and single-cell omics technologies have significantly enhanced the resolution of fascial functional assessment and enabled detailed analysis of cellular heterogeneity and functional compartmentalization. Through single-cell ribonucleic acid sequencing (scRNA-seq), it has been demonstrated that fibroblasts are not a homogeneous population but consist of multiple functional subpopulations [123]. In pathological conditions such as fibrosis, infection, and injury, the primary cell populations driving tissue remodeling can also be identified [41,42,59]. Regarding deep fascial fibrosis, scRNA-seq not only demonstrates that fibroblast and macrophage subpopulations are indispensable for tissue remodeling, but also indicates that widespread activation of fibroblast subpopulations directly leads to the upregulation of various components such as collagen and proteoglycans in the fascial ECM [42]. Studies applying scRNA-seq to fibrotic fascia have demonstrated comprehensive quality control pipelines, including assessment of the number of expressed genes per cell (nFeature_RNA), unique molecular identifiers per cell (nCount_RNA), and the percentage of mitochondrial genes per cell (percent.mt_RNA), with systematic evaluation of correlations among these metrics to ensure data quality. Harmony-based integration of multiple samples has been employed to reduce batch effects and ensure comparability across specimens. It emphasizes standardized procedures from cell isolation and RNA library preparation to data generation, normalization, and analysis, with particular attention to minimizing dissociation-induced transcriptional artifacts through optimized tissue processing protocols [42]. Spatial transcriptomics adds a spatial dimension to these data and enables the analysis of differences in gene expression distribution across different layers, such as the superficial and deep fascia, thereby confirming that there is a clear molecular basis for the functional compartmentalization of the fascia [146]. However, the application of spatial transcriptomics in fascial research remains at an early stage, with a lack of standardized experimental and analytical workflows. Recent studies have established standardized operating procedures and quantitative evaluation frameworks for imaging-based spatial transcriptomics, including assessments of reproducibility, sensitivity, signal-to-noise ratio, and concordance with single-cell transcriptomic profiles [147]. Drawing on these advances, future spatial omics studies of fascia should focus on establishing comprehensive reference datasets encompassing fascial samples from different anatomical regions and pathological conditions, developing standardized protocols for tissue processing, sequencing, and data analysis, and establishing fascia-specific quality-control metrics and reference databases to provide reliable benchmarks for data interpretation.

Moreover, proteomic techniques such as liquid chromatography–tandem mass spectrometry can objectively identify changes in overall protein expression in fascial lesions and have been successfully employed to identify potential biomarkers of adaptive fascial remodeling in acute compartment syndrome [148]. Combined with transcriptomic analysis, these methods allow for the integration of genomic and proteomic information under mechanical or stress conditions, thereby enabling the analysis of the continuous regulatory process from mechanical signals to molecular responses [21]. For the precise quantification of low-abundance molecules or key targets, droplet digital PCR is a reliable method due to its extremely high sensitivity and ability to provide absolute quantification [66]. Traditional techniques such as Western blotting, qPCR, and immunofluorescence continue to play an indispensable role in validating omics results, quantifying target proteins, and analyzing subcellular localization [149,150,151].

Molecular expression and omics technologies provide a solid foundation for the molecular classification of pathology, screening of target molecules, and evaluation of the efficacy of interventions. However, for molecular quantification, the reliability of results depends on appropriate normalization strategies, validated reference genes, antibody specificity, and independent confirmation using complementary approaches. The reproducibility can be improved by applying predefined reporting standards, including detailed descriptions of tissue source, anatomical location, sampling depth, experimental conditions, sequencing parameters, and data-processing pipelines. Due to the high cost of omics technologies and the complexity of data analysis, there is still a long way to go before they become standard techniques. In the future, it may be necessary to adopt standardized reporting guidelines, including the use of validated antibodies, appropriate loading controls, and transparent data presentation, and to combine omics data with techniques such as in vivo signaling pathway tracing, spatial localization, and cellular functional intervention to more comprehensively elucidate the process of pathological fascial remodeling.

#### 4.5.2. Cell Function and Mechanical Stimulation Models

Cell function and mechanical stimulation models used in fascia research provide a key technique for analyzing the mechanisms of mechanical perception and response by simulating the in vivo mechanical microenvironment under controlled conditions. At the tissue level, culturing techniques using fascia grafts allow for direct observation of the dynamic process of spontaneous tissue contraction. Furthermore, combining these techniques with immunofluorescence labeling reveals the causal relationship between the temporal evolution of cell differentiation and the dynamic changes in tissue contraction [54]. The reliability depends on rigorous control of donor/tissue sourcing, isolation protocols, passage number, and culture environments, all of which critically dictate fibroblast phenotype and ECM output [152]. When integrated with in vitro mechanical testing, it is possible to simultaneously quantify the passive mechanical properties of fascial materials like stiffness and load limits, and their active responses to stimuli, thereby validating the fascial contractile capacity and its mechanical remodeling characteristics [1,153]. At the cellular level, primary cultures of isolated fascial fibroblasts offer a valuable research model. With these cultures, cellular responses to mechanical loads, hormones, and drugs can be systematically evaluated. The molecular mechanisms behind ECM synthesis and degradation can also be clarified [19]. Based on this, external shocks, shear stress, and culture systems based on 3D collagen or HA matrices can be introduced. This approach can reveal the cells’ mechanical sensitivity and functional remodeling processes. Moreover, because these conditions more closely resemble the physiological environment, the generalizability of the experimental results is enhanced [21,48]. In these mechanical stimulation models, reproducibility largely depends on the precise characterization of stimulation parameters, including pressure intensity, number of stimulations, strain magnitude, loading frequency, duration, substrate stiffness, and three-dimensional matrix composition [154].

When it comes to dynamic observation, live-cell imaging systems can automatically and continuously monitor the proliferation, migration, or morphological changes in fascial cells after mechanical or chemical stimulation. In this way, data on their dynamic behavior are obtained [48]. Techniques like fluorescence resonance energy transfer microscopy can translate intracellular signaling events, such as transient changes in calcium ion levels or mechanically induced protein interactions, into visible changes in fluorescence signals. Thus, real-time observation of the conversion process between mechanical and biochemical signals becomes possible [21]. Its reproducibility depends on standardized imaging parameters such as acquisition intervals, excitation intensity, temperature control, and rigorous data analysis pipelines. That said, in vitro models lack neural regulation, blood supply, and the body’s complete mechanical environment. The responses seen in these models may differ from those observed in vivo. Therefore, to truly elucidate the dynamic behavior of fascial cells while preserving their physiological integrity, it is necessary to combine in vitro mechanistic studies with in vivo models and clinical observations.

### 4.6. Analysis Based on System Structure and Digital Modeling

#### 4.6.1. Anatomical and Experimental Validation

To understand what fascia does across the whole body, we first need some direct evidence, which is something we obtain from anatomy. Anatomical studies have confirmed that fascia links different regions together through continuous connective tissue, and it also has a rich supply of nerves [155]. That gives us structural confirmation: fascia works as a network, both for transmitting force and integrating sensory information. With that as a foundation, we can then look at how fascia behaves mechanically using cadaver studies under controlled conditions. When researchers apply standardized mechanical tension to cadavers, the relationship between fascial fibers and their force-displacement behavior becomes directly observable. This, in turn, lets us quantify the mechanical relationships among different anatomical layers [1]. Concurrently, cadaveric models can be used to test certain functional ideas. Intervention studies targeting interlaminar structures, such as the separation of sliding surfaces, have demonstrated that the loose connective tissue between fascial layers plays a crucial role in tissue gliding and mechanical coordination [78]. These anatomical findings provide foundational evidence for understanding the biomechanics of fascia and what goes wrong when it does not work properly. However, the translational value of these findings is contingent upon methodological rigor. Standardized protocols for specimen selection, preservation methods, dissection procedures, and measurement techniques are imperative to ensure comparability across studies and to establish a reliable foundation for interpreting fascial dysfunction.

#### 4.6.2. Digital Modeling and Analysis

Digital modeling and analysis have advanced to the point where research on fascial function is no longer just about describing structure. Now it can predict how the system behaves as a whole and tease out causal relationships. The finite element method is currently the most popular way to model continuous media. By feeding parameters not only for fascia but also for bones, tendons, and other tissues, we can simulate how stress is distributed and how force travels when tissue injury or mechanical changes occur [156]. Neuromuscular simulations go further: they incorporate motor control mechanisms and joint connections. That makes it possible to quantify how fascia contributes to gait, stability, and energy efficiency at the whole-body level. Then we can predict motor performance under different structural arrangements [157,158]. By employing the discrete element method and multiscale models, the nonlinear mechanical behavior of fascial materials can be explained through the parameterization of collagen fibers and matrix structures. The application of large-scale models translates the relationship between structural changes, mechanical responses, and functional abnormalities into a computable process [159]. Graph-theory-based network modeling takes a holistic, interconnected view: it sees fascia as a force-transmission network that spans the entire system. That approach helps identify critical structural nodes and evaluate what they mean for system stability. In this way, researchers can quantitatively assess the key functional points within fascia [160]. The predictive accuracy of these methods is contingent upon the quality and biological relevance of the model parameters. Of course, digital modeling cannot replace experiments or physical testing. But it can predict how changes in fascial function affect the whole body’s kinematic and mechanical system without any actual intervention. That gives us a mathematical foundation for mechanism analysis and for designing personalized interventions.

### 4.7. Electrophysiological and Daily Functional Assessments

#### 4.7.1. Electrophysiology

Electrophysiological assessment focuses on bioelectrical characteristics. Its goal is to give us quantitative and objective indicators of how fascia works. When doing local bioimpedance analysis, a low-intensity alternating current is sent through the tissue to measure resistance and impedance. From those measurements, the phase angle is calculated, and it tells us about tissue hydration and whether cell membranes are intact. When these parameters change, they can be sensitive indicators of structural problems such as fascial injury [161]. Bioimpedance tomography can also be used. It produces spatial images and lets us dynamically track average electrical conductivity inside fascial compartments. When external pressure is applied, electrical conductivity goes up progressively. This rise gives an accurate picture of how severe ischemia and edema are in the fascial tissue. In turn, that provides objective, quantitative electrophysiological biomarkers. These biomarkers are useful for early, non-invasive diagnosis of acute compartment syndrome and for regulating interstitial fluid within the fascia [162,163,164]. To ensure reproducible results, standardized protocols must include consistent electrode placement, controlled patient positioning, standardized hydration status, and adherence to established guidelines for bioimpedance measurement procedures. Device calibration using standard procedures with stable references traceable to internationally accredited impedance standards further enhances measurement reproducibility.

Surface electromyography (EMG) has been used in some innovative ways to show that fascia plays an active role in force transmission and neuromuscular control. Traditionally, EMG was only used to assess muscle activity. But because fascia is continuous, researchers can now record unexpected electrical activity in nearby or even farther-away muscles when just one muscle contracts. This gives direct functional evidence that force is transmitted through the fascia [165]. If recording fascial deformation and EMG signals at the same time and then performing a time-series analysis, we can find temporal correlations between the two. That suggests fascia is not only involved in force transmission, but it also helps regulate motor control [166]. Furthermore, when joint torque is kept under certain controlled conditions, EMG signals turn into important indirect indicators. They reflect how the fascial tension system and adjacent muscle groups work together in a synergistic way [167]. So EMG is an effective tool for studying how fascia and muscle function as an integrated system. To ensure reproducibility, standardized protocols must include consistent electrode placement according to the SENIAM project (Surface ElectroMyoGraphy for the Non-Invasive Assessment of Muscles), controlled muscle contraction conditions, standardized normalization methods, and rigorous data processing procedures.

However, most current electrophysiological signals mainly reflect the general electrical properties of tissues. What remains unclear is whether these signals include any active, specific electrical activity coming from the fascia itself. Inside the myofascial system, it is hard to clearly tell which tissue the signals are coming from. We have managed to improve spatial resolution a bit by using invasive electrodes and layered recording. But the ability to separate signals in a specific way is still limited. The reason is that fascia has a thin-layered structure, and electrical signals propagate through it. Going forward, we will need high-resolution electrophysiological techniques to back up and confirm these findings.

#### 4.7.2. Assessment of Activities of Daily Living

The assessment of fascial-related activities in daily life is performed from a clinical perspective. Its goal is to quantify how functional impairments actually affect pain, mobility, and quality of life. So instead of focusing on mechanistic details, the emphasis here is on presenting specific functional outcomes.

What tools do researchers commonly use? Mostly multidimensional clinical scales. Pain intensity is measured with the VAS scale. Local mechanical sensitivity can be assessed objectively using the tender point threshold. To see how much movement is restricted, we measure joint range of motion and use disability scales like the NDI or RMQ. Quality-of-life scales such as the NDI or RMQ help capture the overall functional burden [168,169]. From a functional validation perspective, isokinetic muscle strength testing can reveal the effects of distal interventions on the performance of proximal muscle groups. That gives us indirect evidence of force transmission along fascial chains [170]. Another technique is three-dimensional motion capture, combined with joint kinematic and biomechanical analysis. It has revealed how movements in distal parts can modulate overall movement patterns [171]. Importantly, established clinical outcome scales have been widely validated and routinely applied in musculoskeletal disorders. Their application in fascia-related conditions mainly requires the appropriate selection of disease-specific instruments and standardized assessment procedures. In contrast, objective functional assessments require stricter methodological standardization. Factors such as participant positioning, movement tasks, calibration procedures, testing velocity, and data-processing pipelines should be carefully controlled to minimize measurement variability and improve reproducibility. These techniques focus on the overall performance of fascial function in real-world movement scenarios. But in clinical practice, we still need to interpret them together with the biomechanical assessments, imaging diagnostics, and molecular-level evaluations mentioned earlier. Only then can we achieve a full picture of fascial function.

### 4.8. Challenges in Standardizing Fascial Assessment for Clinical Translation

Despite substantial advances in fascial assessment technologies, their broader application as standardized and clinically interpretable tools remains limited primarily by the lack of globally standardized evaluation frameworks rather than by measurement feasibility itself. First, different studies focus on superficial fascia, deep fascia, myofascial complexes, or region-specific fascial tissues, which differ substantially in structure, biomechanical properties, and pathological characteristics. Thus, measurements obtained using identical techniques may represent different biological targets or pathological entities, limiting cross-study comparability and the development of unified reference standards. Second, measurement heterogeneity arises from variations in acquisition protocols, including patient positioning, tissue loading, and data-processing procedures, as well as differences in equipment, software, and operator expertise. Therefore, high intra- or inter-rater reliability does not necessarily ensure cross-center comparability, which is critical for multicenter validation and consistent clinical interpretation. Furthermore, standardization faces an inherent methodological trade-off: highly controlled protocols enhance reliability but may reduce ecological validity, whereas flexible clinical assessments better capture real-world variability at the expense of reproducibility. This balance remains a fundamental challenge in fascial assessment.

Collectively, the lack of standardized frameworks has hindered the establishment of normative databases, diagnostic thresholds, and interpretation criteria, limiting evidence synthesis, guideline development, and meaningful interpretation at the individual patient level. Without cross-center comparability, measurements obtained in one clinical setting cannot be reliably referenced against external datasets; without longitudinal reliability, they cannot adequately monitor disease progression or treatment response; and without validated thresholds, they cannot determine clinical significance or guide intervention decisions. Therefore, the major challenge is no longer whether fascial properties can be measured, but whether these measurements can be standardized and interpreted sufficiently to support consistent clinical decision-making across healthcare settings.

Future efforts must shift from proliferating isolated technologies toward establishing an internationally harmonized framework for fascial evaluation. This framework should systematically integrate standardized anatomical definitions, acquisition protocols, reporting guidelines, normative databases, and clinically validated thresholds. Implementing such a framework will require multicenter collaborative validation studies, and, where appropriate, automated image analysis and artificial intelligence-assisted workflows to reduce operator-dependent variability. Concurrently, an international expert consensus involving anatomists, imaging specialists, biomechanical researchers, and clinicians is essential to bridge terminological divides. Ultimately, such a unified ecosystem will enable reliable cross-center data sharing, improve measurement reproducibility, and provide a robust foundation for evidence generation and future clinical translation of fascial assessment.

**Figure 3:**
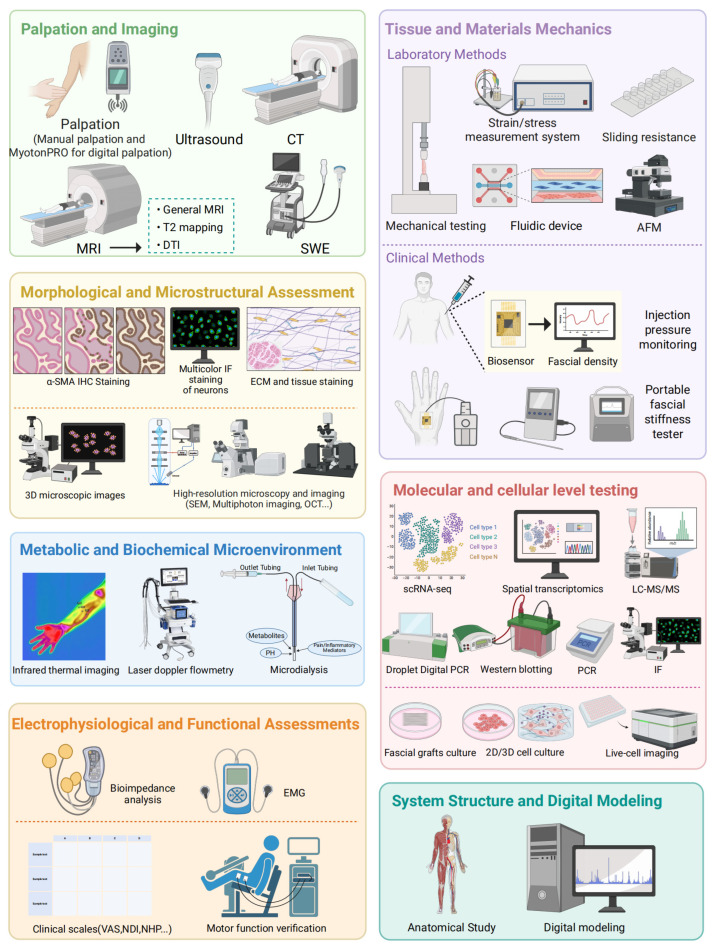
Schematic Diagram of Current Technical Methods for Fascia Function Assessment and Diagnosis. This figure covers seven key aspects of fascial functional assessment: palpation and imaging, tissue and material mechanics, morphological and microstructural evaluation, metabolic and biochemical microenvironment, molecular and cellular level testing, electrophysiological and functional assessment, and systemic structure and digital modeling. It brings together a wide range of technical approaches that span from the macroscopic to the nanoscale, from static structure to dynamic function, and from empirical palpation to quantitative modeling. This provides researchers with a systematic reference for understanding the diagnostic tools and techniques used in the field of fascial function assessment. Created in https://BioRender.com.

In summary, fascial function assessment is moving away from just describing structure and symptoms in one dimension. It is becoming a multidimensional system that brings together mechanics, structure, metabolism, electrophysiology, and molecular mechanisms (Figure 3). Each method has its own focus, but using them together gives us a comprehensive view of how the fascia is working. That not only helps us better understand the pathological mechanisms behind fascial-origin disorders, but also provides an objective and multidimensional basis for clinical diagnosis, treatment decisions, and evaluation of treatment outcomes. Looking ahead, the future of fascia function assessment will show three major trends: technological integration, dynamic and intelligent approaches, and simplification and standardization centered on clinical practice. This will enable us to accurately assess the functional state of the fascia and reliably determine whether an intervention is effective. In the end, complex test results can truly and effectively serve clinical decision-making.

## 5. Regulation of Fascial Function: Treatments and Methods

**Figure 4:**
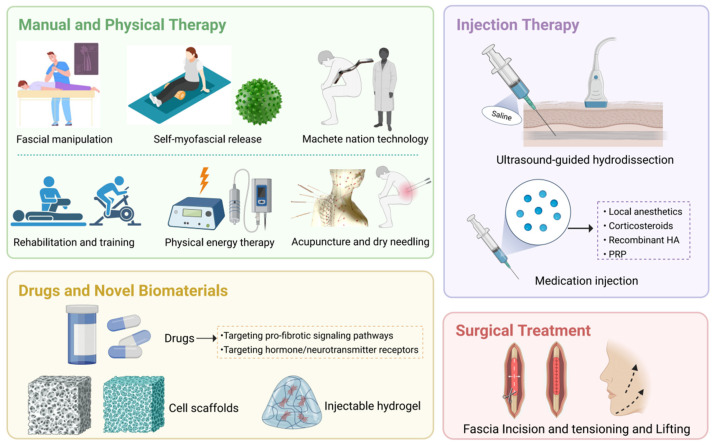
Schematic diagram of methods and techniques for fascial function regulation. This figure covers four areas: manual and physical therapy, injection therapy, surgical treatment, and medications and novel biomaterials, illustrating multi-level fascial function regulation techniques that range from mechanical intervention and chemical modulation to structural reconstruction. This provides a reference for researchers to learn about the methods and techniques used in the treatment of fascia-related disorders. Created in https://BioRender.com.

Various methods have been developed to regulate fascial function, ranging from manual and physical therapies to injections, surgeries, and the emerging biomaterial-based approaches (Figure 4).

### 5.1. Manual and Physical Therapy

#### 5.1.1. Fascial Manipulation (FM)

FM is an intervention that applies external mechanical stimulation. Pressure, traction, or vibration is delivered to subcutaneous tissues. Through this approach, the mechanical state and sensory input of the fascial system are adjusted, with the aim of restoring normal fascial movement and its ability to modulate pain. Its use is not limited to the musculoskeletal system; it also extends to the visceral fascia. By improving how tissues glide and how tension is distributed, FM can help modulate organ function and postural stability [172,173]. From a technical standpoint, different patterns of stimulation trigger different physiological responses. For example, slow, deep pressure mainly promotes tissue relaxation. High-speed vibration, on the other hand, helps with sensory re-education and neuromodulation [28]. Given the inherent characteristics of myofascial chains, treatment should focus on primary tension areas. That way, mechanical forces can be redistributed across different regions, restoring systemic balance and making the therapeutic outcomes more stable and durable [174]. A systematic review including 12 randomized controlled trials (RCTs) and 4 observational studies published between 2015 and 2025 reported that FM produced significant improvements in pain reduction (20–50%), range of motion (15–35% absolute improvement), and functional recovery across various musculoskeletal conditions [175]. In a specific clinical setting, an RCT involving 88 patients with patellar tendinopathy demonstrated that FM combined with eccentric exercise and static stretching resulted in significant improvements at 4 weeks, 3 months, 6 months, and 1 year of follow-up. Moreover, the FM group required fewer treatment sessions and achieved higher patient satisfaction compared with the exercise-only group [176]. Although FM has shown beneficial effects on fascial pain conditions, recent meta-analyses have rated the overall certainty of evidence as low to very low by GRADE assessment because of small sample sizes, methodological heterogeneity, and insufficient blinding procedures [177]. Its underlying mechanisms primarily involve synergistic regulation of biomechanics, the nervous system, and the local microenvironment. More specifically: improving fascial glide, modulating sensory input, and optimizing local metabolic states [177,178]. Future research should focus on optimizing treatment parameters according to disease characteristics and individual treatment responses, while promoting the standardization of intervention protocols. In addition, large-scale randomized controlled trials incorporating objective assessments of fascial structure and function are needed to establish quantitative efficacy evaluation systems, improve the reliability and reproducibility of clinical findings, and provide stronger evidence-based recommendations for fascia-targeted therapies.

#### 5.1.2. Myofascial Release

Myofascial release is currently one of the most common physical intervention methods for improving myofascial dysfunction. Its goal is to restore fascial glide through external mechanical stimulation and adjust tension distribution. And in doing so, influence neuromuscular control and systemic functional integration. Based on how it is performed, this technique can be divided into two types: self-myofascial release and professional manual therapy. Self-myofascial release has become the most widely used at-home technique in recent years. It is usually performed with tools like foam rollers or massage balls. By rolling over the fascia, we apply pressure roughly equal to body weight, giving the tissue sustained mechanical stimulation. Research shows that this approach improves local tissue flexibility and glide, helps ease post-exercise muscle soreness, and increases joint range of motion without hurting athletic performance [179]. Self-myofascial release combined with Pilates training demonstrated additive effects in reducing pain and muscle stiffness in patients with chronic low back pain [180]. The effects are not just local; they can also reach distal regions along the fascial chain [15,181]. The distant release technique added to the exercise training on flexibility, muscular endurance, and dynamic balance was greater than the exercise training alone [182]. However, self-myofascial release with foam rollers is supported by moderate evidence for acute improvements in flexibility and range of motion, but lacks consistent evidence for pain modulation or tissue-level fascial changes [183]. Thus, for self-myofascial release techniques, the evidence is more mixed and requires careful interpretation.

On the professional side, techniques like machete nation technology are typically performed by therapists. That allows for more precise control of the application points and intensity of force. Clinical studies have shown that when combined with active functional training, these techniques can relieve pain, improve muscle endurance, and enhance overall functional status more effectively [184]. Several systematic reviews and meta-analyses have demonstrated the clinical effectiveness of myofascial release across diverse patient populations. A meta-analysis in patients with fibromyalgia showed that myofascial release significantly reduced pain (standardized mean difference = −1.16, *p* = 0.0003) [185]. Another study reported a higher level of evidence for pain relief with myofascial release compared with several other interventions [173]. A study that assessed methodological quality using the PEDro scale and evaluated the certainty of evidence according to the GRADE framework found that myofascial release produced a moderate effect size for improving joint ROM [186]. Even though myofascial release comes in many forms, its basic goals are generally similar. Looking ahead, more data from high-quality randomized controlled trials will be needed. That would help improve the precision of how these techniques are applied and give them a stronger scientific foundation.

#### 5.1.3. Rehabilitation and Physical Training

Rehabilitation and physical training are two active strategies for regulating fascial function. They also help promote structural and functional adaptation in the fascia. Studies have shown that moderate physical activity encourages HA metabolism and blood circulation within tissues. That, in turn, helps maintain the gliding ability between different fascial layers. Conversely, prolonged inactivity or excessive loading increases fascial viscosity. That leads to reduced gliding ability and poorer function [40]. A systematic review and meta-analysis of 24 RCTs indicated that exercise programs may be an effective approach for improving pain intensity, pressure pain threshold, and ROM in patients with myofascial trigger points [187]. Based on the theory of fascial continuity, modern rehabilitation is increasingly turning to holistic training models that focus on myofascial chains. By doing stretching and strengthening exercises aimed at specific functional chains, you can optimize whole-body force distribution and mechanical efficiency. This kind of training is now widely used in pain management and for boosting athletic performance [18]. Clinical data also support the benefits of multimodal rehabilitation. Randomized controlled trials have found that systematic rehab programs that include physical training work better than a single therapy alone. They are more effective at relieving pain, improving function, and reducing fascial thickening [188]. Similarly, systemic rehabilitation emphasizes examining abnormal stress from the perspective of whole-body biomechanics. Then, tension is redistributed through movement to improve fascial dysfunction [189]. A systematic review and meta-analysis of 14 RCTs (N = 734) demonstrated that exercise rehabilitation was moderately effective as an adjunctive intervention for the clinical management of myofascial trigger points; however, further large-scale, high-quality studies are required to confirm these findings [190]. Right now, a clinical challenge remains unsolved: how to determine the most suitable type, intensity, and frequency of exercise for patients who have very different clinical presentations and are at different stages of their disease. Going forward, rehabilitation strategies need to be based on precise assessments of each person’s fascial status. We have to move away from empirical training and toward personalized, precise exercise prescriptions.

#### 5.1.4. Physical Energy Therapy

High-frequency thermotherapy and extracorporeal shock wave therapy are both physical therapy techniques. They regulate fascial function through energy input. High-frequency thermotherapy primarily improves local blood circulation and metabolism by elevating tissue temperature. It, in turn, helps create a better environment for repair. But to really boost structural remodeling, you usually need to combine this therapy with mechanical interventions [96]. Extracorporeal shock wave therapy works in a more multidimensional way. Supporting its efficacy, a 2024 meta-analysis (16 RCTs, n = 1121) utilizing rigorous PEDro and Cochrane assessments confirmed that extracorporeal shock wave therapy yields better mid-to-long-term outcomes than corticosteroid injections for plantar fasciitis [191]. Another RCT involving 64 patients with myofascial pain syndrome demonstrated that focused extracorporeal shockwave therapy significantly reduced the stiffness of both myofascial trigger points and the surrounding fascia, while effectively alleviating pain and functional impairment [192]. For one thing, it modulates afferent signals coming from peripheral sensory nerves, which helps block pain signal transmission. For another, it acts directly on fascial fibroblasts, triggering remodeling and regeneration processes inside the fascial ECM [36,193]. This shows that shock wave therapy can actively regulate how tissues repair themselves. Both techniques do more than just physically impact the fascia. They also exert effects by influencing the local microenvironment and how cells respond. That makes them a good clinical option. In summary, extracorporeal shockwave therapy currently has established supporting evidence, as demonstrated by multiple clinical analyses. In contrast, the evidence supporting high-frequency thermotherapy remains limited, and further studies are required to validate its efficacy. That said, we still need to choose the most suitable technique based on the specific pathological target in practice. And it is worth looking for combined strategies that synergize with other mechanical interventions.

#### 5.1.5. Acupuncture and Dry Needling

Acupuncture and dry needling are two common therapeutic techniques. They come from different theoretical backgrounds, but both use “needles” as a way to regulate fascial function. Dry needling is a modern Western medicine technique. Its goal is to relieve muscle and fascial pain by inserting needles into trigger points or painful areas. Acupuncture, on the other hand, comes from traditional Chinese medicine. It aims to regulate overall body functions by inserting needles into specific acupoints of meridians. Both techniques can produce a series of biological effects through mechanical stimulation of fascial tissue.

For conditions associated with myofascial pain, studies have confirmed that dry needling can improve local structural and biomechanical characteristics [194,195]. Clinical evidence has also demonstrated potential benefits. For example, in plantar fasciitis, dry needling reduces fascial thickness and improves its elasticity, thereby restoring biomechanical continuity while alleviating pain and functional impairment [195,196]. Supporting these findings, a 2024 systematic review and meta-analysis of 12 RCTs involving 781 patients demonstrated that dry needling combined with conventional treatments significantly reduced pain (95% CI −2.12 to −1.76, *p* < 0.0001) and improved foot function (95% CI −12.57 to −3.58, *p* = 0.004) compared with conventional treatments alone [197]. However, broader evidence remains limited. Network meta-analyses and GRADE-based evidence synthesis have shown no clear superiority of dry needling over manual therapy or corticosteroid injection for myofascial pain conditions [198], with low certainty of evidence due to methodological limitations and protocol heterogeneity [199]. When combined with physical training, its therapeutic effects are further enhanced, indicating a synergistic relationship with mechanical load regulation [200].

The evidence base for acupuncture in fascial and myofascial conditions has grown substantially. For myofascial pain syndrome, a 2024 systematic review and meta-analysis of 10 RCTs involving 852 patients demonstrated that acupuncture was significantly more effective than control interventions [201]. However, publication bias was identified for VAS scores, which may affect the reliability of some conclusions [202]. The effects of acupuncture have also been confirmed not to be limited to local pain relief but rather exert regulatory effects through multiple pathways. Fascial fibroblasts are considered the main target cells. The mechanical stimulation caused by acupuncture can activate these cells, thereby promoting the release of bioactive substances such as adenosine and HA, and forming an interaction network with immune cells, thereby producing analgesic and repair effects [203]. Building on this, an enhanced form of acupuncture called electroacupuncture exerts anti-inflammatory effects by modulating signaling pathways such as TGF-β1/SMAD family member 2, thereby inhibiting excessive fibroblast activation and promoting fascial regeneration [149]. These mechanistic findings are consistent with clinical observations of improved fascial tension following acupuncture interventions [204], making acupuncture a highly promising and extremely useful intervention in the field of traditional Chinese medicine. Further high-quality studies are needed to optimize acupuncture protocols and clarify their clinical efficacy in fascial dysfunction. Future research should prioritize standardized intervention parameters, including acupoint selection, needling depth, manipulation techniques, treatment frequency, direct comparisons among different acupuncture modalities, characterization of dose–response relationships, and long-term evaluation of therapeutic sustainability, in order to optimize the precise application of these two therapies.

### 5.2. Injection Therapy

Fascial injection therapy is an interventional technique aimed at treating fascial dysfunction. It primarily includes fascial blocks, ultrasound-guided fluid aspiration, hyaluronidase injections, and platelet-rich plasma (PRP) injections. These techniques work in different ways: some use pharmacological modulation, others rely on mechanical relief, matrix remodeling, or tissue regeneration. Their shared goal is to regulate fascial nerve sensitivity, gliding ability, and the state of the ECM.

Fascial block therapy takes advantage of the low diffusion resistance found in the fascial space. Because of that, local anesthetics can spread into loose connective tissue and the fluid matrix. This allows for selective inhibition of nociceptors and their neural innervation [205]. Under the influence of pressure gradients, the drug diffuses through molecular clustering, producing an interlaminar analgesic effect [206]. The efficacy depends on fascial thickness, the viscoelasticity of HA, and the degree of fibrosis [19]. So it can be inferred that structural heterogeneity is a key reason why outcomes vary. Although pain relief has been shown, there is still a risk of systemic toxicity if the dose is too high. That is why dosage and procedural protocols need to be strictly controlled [207].

Ultrasound-guided hydrodissection precisely delivers fluids such as saline between the fascial layers. It creates both mechanical separation and hydrostatic expansion [208]. Its effectiveness is manifested in two ways: it dissolves adhesions and restores gliding ability, and the fluid pressure and tissue deformation caused by the injection can stimulate and regulate the nerve endings within the fascia. That gives the central nervous system therapeutic sensory input [4,78]. However, evidence for fascial plane blocks and intrafascial injections is mainly accumulating in perioperative settings. For example, ultrasound-guided fascial plane blocks have been shown to provide effective and safe postoperative analgesia [209], whereas evidence supporting their application for fascial dysfunction itself remains limited.

Corticosteroids have also long been used for short-term pain relief in cases of fascial inflammation and tendon disorders. However, they reduce the mechanical strength of collagen. So if they are used long-term or repeatedly, there may be a risk of structural damage [57]. Injection of recombinant HA is a chemical intervention. It breaks down abnormally aggregated HA, reducing the viscosity and density of the matrix. That helps restore the fascia’s ability to glide and transmit force [210]. Subfascial PRP injections focus more on modulating regenerative processes. PRP is rich in growth factors. It can activate endogenous stem cells, stimulate remodeling of the ECM outside the fascia, and regulate inflammatory responses. All of that promotes tissue repair. This suggests that PRP could be useful for certain fascia-related painful diseases [211]. Across short-, intermediate-, and long-term follow-up periods, corticosteroid injections demonstrated the greatest short-term functional improvement, whereas PRP injections were associated with sustained improvements in function and reductions in plantar fascia thickness over the long term [212]. A randomized double-blind study further showed that hyaluronidase combined with lidocaine injection provided superior therapeutic effects compared with lidocaine alone in patients with myofascial pain syndrome [213]. However, the overall certainty of evidence from these studies remains low to moderate, and larger, high-quality trials are required to validate these findings. Future research should focus on determining the optimal indications, duration of therapeutic effects, and combination treatment strategies for different injection-based interventions.

### 5.3. Surgical Treatment

The main goal of surgical treatment is to restore fascial function by rebuilding its structure. Surgery is usually considered when conservative treatments have not worked, or when there is obvious structural damage that needs fixing. Fascial incision is an emergency procedure used to treat acute conditions that threaten limb circulation, such as acute compartment syndrome. When pressure inside a compartment becomes abnormally high, a vertical incision can be made to relieve that tension. This quickly restores blood flow to the tissue and stops ischemic injury from becoming worse. Without this step, it is hard to preserve nerve and muscle function [214]. A systematic review including 45 studies (38 case reports and 7 cohort studies) identified fascial release through fasciotomy as the primary surgical intervention. However, no consensus has been reached regarding the optimal number or anatomical location of incisions, and the substantial heterogeneity among studies has precluded a meta-analysis of the risks associated with different incision techniques [215]. After surgery, how the fascia is closed matters. Techniques like direct suturing or skin flap grafting will affect local mechanical repair and the functional prognosis. In fact, the choice of closure technique is a decisive factor in how well the patient recovers [216]. Fascial suspension and lifting procedures are widely used in cosmetic and reconstructive surgery. They directly restore fascial tension and support by folding, suspending, or excising excess fascia. That helps reverse age-related fascial laxity and loss of support [131]. Due to variations in fascial structure across different regions, surgical strategies typically need to be tailored to specific cases. The goal is to restore the normal interface and mechanical connection between the fascia and subcutaneous fat tissue, thereby achieving long-term stability in fascial structure and functional reconstruction. Most available evidence is derived from case series or expert opinions rather than rigorous RCTs or large-scale cohort studies. The lack of standardized surgical protocols and objective outcome assessment systems remains a major limitation.

### 5.4. Drugs and Novel Biomaterials Targeting Fascia

#### 5.4.1. Drugs

Pharmacological treatments targeting the fascia are based on its specific molecular expression profile and aim to modulate specific signaling pathways associated with fascial dysfunction. Drugs that target pro-fibrotic signaling pathways represent an effective strategy for inhibiting pathological remodeling processes. The renin-angiotensin system receptors found in the fascia provide direct targets for anti-fibrotic therapy. Clinically widely used angiotensin type 1 receptor antagonists such as irbesartan, losartan, and valsartan offer potential intervention pathways for alleviating fascial fibrosis and stiffness associated with hypertension and glucose metabolism disorders [66,99]. Ongoing clinical trials are currently evaluating losartan for conditions such as joint fibrosis and skeletal muscle disorders, as well as comparing the anti-fibrotic efficacy of different angiotensin II receptor blockers [217]. However, despite relatively strong preclinical evidence supporting the potential of anti-fibrotic agents in regulating fascial remodeling, clinical trials targeting fascia-specific indications remain at an early stage. Drugs targeting hormone and neurotransmitter receptors also provide new approaches for regulating fascial homeostasis and treating pain. Recombinant relaxin and its analogs, by inhibiting fibroblast activation, may serve as novel active drugs for treating fibrotic conditions such as plantar fasciitis. Meanwhile, modulators that act on fascial cannabinoid 1 (CB1)/CB2 receptors, such as cannabidiol, may represent a new therapeutic option for chronic myofascial pain due to their anti-inflammatory and local analgesic effects [5,39]. Although basic research has identified potential pharmacological targets within fascial tissues, current evidence remains insufficient to establish definitive clinical benefits. Further high-quality randomized controlled trials are required to validate their therapeutic efficacy and clinical applicability. Targeting specific molecules within the fascia with drugs might offer a new way to treat fascial disorders.

Translating these preclinical pharmacological strategies into clinical practice holds promise for personalized treatment of fascial dysfunctions, as studies have revealed substantial molecular and biomechanical heterogeneity among fibrotic, inflammatory, and mechanically altered fascial phenotypes. This transition may shift fascia therapy from empirical approaches toward phenotype-guided interventions, matching anti-fibrotic or pain-modulating therapies to individual pathological profiles. Moreover, integrating molecular profiling, advanced imaging, and quantitative biomechanical assessments could facilitate treatment monitoring and individualized therapeutic adjustment. To realize this potential, future efforts should prioritize establishing clinically accessible biomarkers for patient stratification and conducting mechanism-driven stratified clinical trials to validate efficacy in defined subgroups, ultimately facilitating the clinical translation of fascia-targeted therapies.

#### 5.4.2. Biomaterials and Tissue Engineering

Biomaterials and tissue engineering are bringing new strategies for functional reconstruction of the fascia. Their role goes beyond just replacing damaged tissue structurally. The goal is to build biomimetic scaffolds that can mimic the natural microenvironment of fascia. These scaffolds should be able to control targeted cell migration, promote functional tissue integration, and ultimately restore the fascia’s mechanical and physiological functions.

With that in mind, some researchers have put forward the following hypotheses [9]. Scaffolds made from a decellularized fascia matrix derived from natural fascia retain most of the ECM components. They also have low immunogenicity. Because of that, they can serve as ideal carriers for delivering ECM and for promoting remodeling and integration of the host tissue. Then there are bionic synthetic scaffolds, such as nanofiber scaffolds made by electrospinning, or oriented collagen fiber scaffolds. These can precisely copy the nanoscale topological structure and collagen fiber orientation found in natural fascia. By doing so, they allow targeted control over how fascial fibroblasts migrate. That promotes ordered regeneration and reduces the formation of disorganized scar tissue. Injectable hydrogels are another option. They have excellent in situ plasticity, which makes them ideal drug delivery carriers. They can carry cytokines, drugs, or stem cells, and then be injected into the fascial layer. There, they help repair hard-to-heal injuries like chronic wounds by modulating the local immune microenvironment. Cell scaffold technology is also worth mentioning. It enables the combination of mesenchymal stem cells with scaffolds and delivers them directly to the fascial layer. This approach not only harnesses the regenerative capacity of transplanted cells but also effectively mobilizes the repair potential of precursor cells already inside the fascia. The result is to achieve localized regeneration. Although these material strategies show great promise, most of them are still in the early stages of exploration and validation. While the path from concept to application is filled with challenges, these ideas nevertheless open up promising new directions for the future of functional fascia regeneration.

If these emerging biomaterial-based strategies can be successfully translated into clinical practice, they may provide more personalized therapeutic approaches for fascial repair. This transition is expected to occur progressively: initial clinical applications may rely on standardized biomaterial products that have been validated for specific indications; subsequently, advances integrating quantitative fascial assessment, imaging evaluation, and biomechanical analysis may enable more precise matching of biomaterials with individual fascial pathological conditions. Ultimately, future regenerative strategies may evolve toward patient-specific designs, in which scaffold composition, bioactive signals, and therapeutic components are customized according to individual fascial characteristics. For example, mechanically reinforced scaffolds may be applied for large structural defects, drug-releasing hydrogels may be utilized for chronic fibrotic lesions, and cell-loaded constructs may be developed for patients with impaired regenerative capacity. By integrating objective fascial phenotyping with personalized regenerative strategies, these advances may shift clinical management from empirical reconstruction toward mechanism-based and patient-specific treatment approaches for fascial dysfunctions.

## 6. Conclusions

This article focuses on the functions of fascia, providing a systematic analysis and discussion that covers aspects ranging from physiological characteristics to functional disorders, and innovatively integrates assessment and intervention methods for fascia. It highlights the central role of fascia in musculoskeletal pain and functional disorders, noting that fascia serves as both a cause and a key diagnostic factor, as it is also the target for treatment.

However, fascia research as a whole remains in a rapidly developing stage and has not yet reached maturity. First, certain fields such as fascia biology and endocrinology have not been systematically elucidated, and the regulatory mechanisms of their molecular networks and microenvironment remain unclear. Second, in clinical practice, the focus remains on muscles, and the pathological significance of fascia itself, as well as the value of fascia-targeted interventions, has not yet been fully recognized. Another issue is that a quantitative assessment system for fascial function has not yet been established. Across different studies, there are big differences in how fascia is defined, how it is measured, and what outcome indicators are used. That makes it hard to integrate or compare the evidence. Also, there is a noticeable gap between basic research and clinical practice. On one hand, collecting human tissue samples is limited by ethical constraints. On the other hand, animal models struggle to accurately mimic the biomechanical properties of human fascia. So the depth of mechanistic research is restricted. The mechanisms underlying the interaction between the pathogenesis of myofascial disorders and psychological stress and emotional states have not yet been fully elucidated. That needs further research within a mind–body medicine framework. The causes of referred pain and how distal interventions work are still debated. There is no direct evidence to say whether these effects come from neural pathways, mechanical actions, or a combination of both. In addition, this review mainly focuses on the musculoskeletal fascia. It does not systematically look at visceral fascia. The role of visceral fascia in regulating organ function and in the broader systemic network is something that needs further investigation.

Looking ahead, future research should aim to build a multimodal, precision-oriented fascial science. We need stronger mechanistic studies that focus on the fascia itself. Cross-validation between molecular biology and biomechanics should be encouraged. In clinical practice, we should push for standardized, quantifiable assessment tools. Rigorous clinical trials can then provide high-level evidence to guide treatment decisions related to fascia. With advances in computer modeling and artificial intelligence, mechanistic simulations and image recognition based on fascial networks could become important tools for auxiliary diagnosis and for personalized treatment. In short, only with the help of new technologies and methods, by establishing effective collaboration between basic research and clinical translation, and by shifting fascia research from fragmented exploration to a unified framework, can we truly advance fascia-centered precision diagnosis and treatment.

## Data Availability

No new data were created or analyzed in this study. Data sharing is not applicable to this article.

## Abbreviations

The following abbreviations are used in this manuscript:

HA	hyaluronic acid
α-SMA	alpha-smooth muscle actin
ECM	extracellular matrix
TGF-β1	transforming growth factor-β1
MRI	magnetic resonance imaging
CT	computed tomography
SWE	shear-wave elastography
AFM	atomic force microscopy
OCT	optical coherence tomography
scRNA-seq	single-cell ribonucleic acid sequencing
EMG	surface electromyography
FM	fascial manipulation
PRP	platelet-rich plasma
CB	cannabinoid
ICC	intraclass correlation coefficient
RCT	randomized controlled trial
